# From the brain cell atlas to precision neurology: a review of the application of AI-driven multi-omics in brain science

**DOI:** 10.1093/gigascience/giag075

**Published:** 2026-06-23

**Authors:** Youzhe He, Yanrong Wei, Jingxi Zhi, Chunyu Huang, Lei Han, Shiping Liu, Lifang Wang

**Affiliations:** College of Life Sciences, University of Chinese Academy of Sciences, Beijing 100049, China; BGI Research, Hangzhou 310030, China; College of Life Sciences, University of Chinese Academy of Sciences, Beijing 100049, China; BGI Research, Hangzhou 310030, China; BGI Research, Hangzhou 310030, China; School of Life Science, Hangzhou Institute for Advanced Study, University of Chinese Academy of Sciences, Hangzhou 310030, China; College of Life Sciences, University of Chinese Academy of Sciences, Beijing 100049, China; BGI Research, Hangzhou 310030, China; BGI Research, Shenzhen 518083, China; Key Laboratory of Brain Cell Mapping of Zhejiang Province, BGI Research, Hangzhou 310030, China; College of Life Sciences, University of Chinese Academy of Sciences, Beijing 100049, China; Key Laboratory of Spatial Omics of Zhejiang Province, BGI Research, Hangzhou 310030, China; BGI Research, Hangzhou 310030, China; Key Laboratory of Spatial Omics of Zhejiang Province, BGI Research, Hangzhou 310030, China

**Keywords:** Brain cell atlas, artificial intelligence, multi-omics, precision neurology

## Abstract

Recent advances in multi-omics technologies have catalyzed the construction of comprehensive brain cell atlases, providing essential data foundations for artificial intelligence (AI)-driven analyses in precision neurology. This review systematically examines how the integration of AI with single-cell multi-omics and spatial multi-omics advances the resolution in deciphering brain cellular architecture across health and disease states. Through systematic evaluation of multi-omics datasets from neurodegenerative, psychiatric, and neurodevelopmental disorders, we demonstrate how AI facilitates disease subtype stratification, biomarker discovery, and therapeutic target identification. We critically address translational challenges, including data standardization, model interpretability, and regulatory frameworks for clinical implementation. Notably, the establishment of the International Consortium for Primate Brain Mapping in 2025 exemplifies ongoing global collaborative efforts toward systematic multi-omics atlas construction across species and disease states. This synthesis underscores a paradigm shift toward AI-enabled, mechanism-driven analyses, ultimately positioning precision neurology as a realizable framework for individualized diagnosis and targeted interventions in complex brain disorders.

## Introduction: a paradigm shift in brain research

The brain, as one of the most intricate organs, comprises approximately 86 billion neurons interconnected into sophisticated networks [1, 2]. Understanding its architecture and functional dynamics is essential not only for deciphering the substrates of higher-order cognitive processes such as consciousness and memory, but also for elucidating the pathophysiological mechanisms underpinning neurological disorders, including Alzheimer’s and Parkinson’s diseases (PD) and psychiatric disorders [3, 4]. Brain atlases, which provide reference frameworks for mapping the brain’s architecture, connectivity, and function, have become fundamental resources for understanding brain organization and for supporting neuroscience research [5–9]. Traditional atlases, largely based on macroscopic imaging such as magnetic resonance imaging (MRI) and static histology, capture population-level templates and common brain features. However, they fail to reflect individual variability, developmental dynamics, or the complex pathological essence of diseases [10, 11].

We are now witnessing a profound paradigm shift, moving from purely descriptive mapping toward mechanistic understanding and precision neurology [11, 12]. This shift is driven by 2 complementary forces: (1) emerging technologies as core drivers of mechanistic insight. Single-cell and spatial multi-omics technologies enable unprecedented resolution in dissecting cellular and molecular heterogeneity. For example, snRNA-seq identifies distinct cell types and transcriptional states but loses spatial context, whereas spatial transcriptomic methods such as Stereo-seq retain spatial organization at the expense of whole-transcriptome coverage. Multi-modal integration of these datasets enables the comprehensive mapping of cell types and circuit-level interactions that underlie function and disease [13–15]. AI, particularly deep learning, further enhances this integration by detecting patterns imperceptible to humans, predicting cell states, and inferring regulatory relationships from complex multi-dimensional data [16–18]. Complementary cross-species comparative studies and multi-omics atlas construction across model organisms provide evolutionary perspectives that clarify conserved mechanisms and accelerate translational insights into human brain disorders [19–24]. Collectively, these technological advances support precision medicine approaches, allowing integration of an individual’s genetic background, multi-modal atlas data, and clinical phenotypes for disease prediction, subtype stratification, and therapeutic guidance [25]. (2) Evolution of research goals from description to mechanism. Alongside technological advances, the objectives of brain atlas research have fundamentally evolved. The field is moving beyond asking “what it looks like” to probing “how it works” and “how it goes awry” [26]. Achieving this requires dynamic, comprehensive healthy brain atlases as a reference baseline against which dysregulation of genes, circuits, and networks can be precisely measured in complex disorders such as schizophrenia, depression, and Alzheimer’s disease (AD). The ultimate aim is to translate these mechanistic insights into clinical interventions [27, 28]. By identifying key mechanistic nodes through basic research and leveraging AI-driven biomarker discovery, researchers can accelerate development of novel neuromodulation technologies, drug targets, and small-molecule therapeutics, bridging the gap from bench to bedside [29].

**Figure 1:**
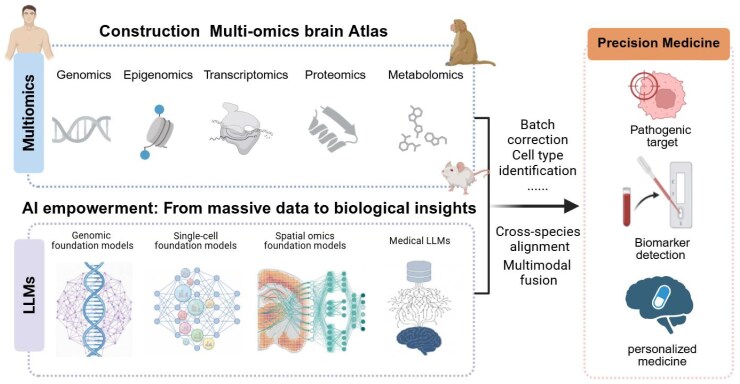
From multi-omics to precision medicine: an AI-enabled brain atlas framework. This figure illustrates the integrative workflow from multi-omics data generation to precision neurology applications. The framework encompasses 3 major components: (1) construction of multi-omics brain atlases, where genomics, epigenomics, transcriptomics, proteomics, and metabolomics data are systematically collected across model organisms (human, non-human primates, mouse, etc.) to generate comprehensive molecular maps; (2) advanced AI models, including genomic foundation models, single-cell foundation models, spatial omics foundation models, and medical LLMs, are leveraged to perform batch correction, cell type identification, cross-species alignment, and multimodal data fusion, converting large-scale data into actionable biological knowledge; and (3) precision medicine applications, where AI-driven analysis enables identification of pathogenic targets, detection of disease-specific biomarkers, and development of personalized therapeutic strategies. This integrated framework exemplifies the paradigm shift from descriptive atlas construction to mechanism-driven precision interventions, ultimately translating multi-dimensional molecular insights into clinically actionable solutions for brain disorders.

This review systematically examines how AI and multi-omics are reshaping brain atlas construction; evaluates how cross-species integration informs human brain disease mechanisms; and envisions how atlas-based personalized diagnosis and therapy can move from concept to reality in the era of precision medicine. We also address critical challenges, including data standardization, computational resource allocation, and ethical considerations, providing a strategic roadmap for advancing the field (Fig. 1).

## Multi-omics mapping technology platform

**Figure 2:**
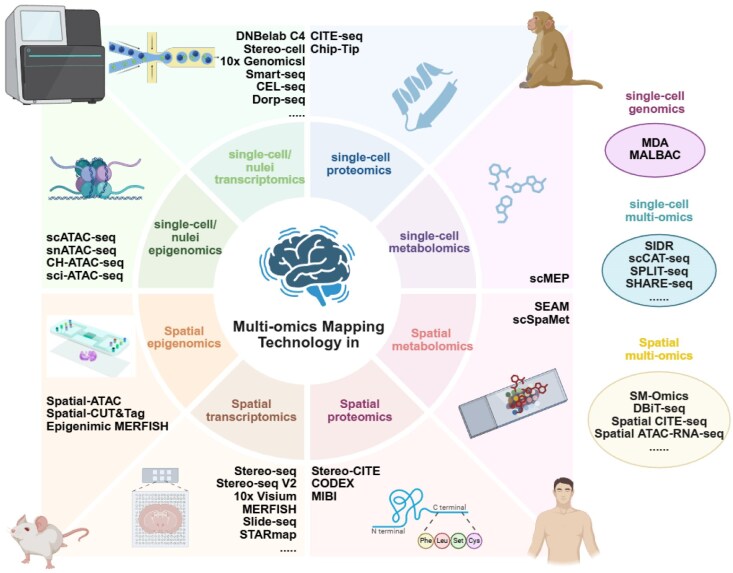
Multi-omics technology in brain science research (see Table 1). This figure illustrates the comprehensive multi-omics technology ecosystem for brain cell atlas construction. Single-cell/nuclei technologies enable molecular profiling of individual cells across transcriptomics (e.g., 10x Genomics, DNBelab C4, Stereo-cell), epigenomics (scATAC-seq, snATAC-seq), proteomics (e.g., CITE-seq, Chip-Tip), metabolomics (e.g., scMEP), and genomics (MDA, MALBAC). Single-cell multi-omics integration platforms (e.g., SIDR, SPLIT-seq, PHAGE-ATAC) simultaneously capture multiple molecular layers from the same cell. Spatial technologies preserve native tissue architecture while mapping molecular information, including spatial transcriptomics (e.g., Stereo-seq, 10x Visium, MERFISH), spatial proteomics (e.g., Stereo-CITE, CODEX, MIBI), spatial epigenomics (e.g., Spatial-ATAC, Epigenomic MERFISH), and spatial metabolomics (e.g., SEAM). Spatial multi-omics platforms (e.g., SM-Omics, Spatial CITE-seq) enable concurrent multi-modal detection with spatial resolution. Model organisms depicted include mouse, non-human primates, and humans, representing the cross-species comparative framework essential for translational brain research.

Breakthroughs in neuroscience have historically been contingent upon technological innovation. The development and maturation of single-cell multi-omics technologies have enabled systematic investigation of brain complexity at cellular resolution. These technologies permit simultaneous profiling of transcriptomic, epigenomic, proteomic, and genomic features within individual cells, thereby facilitating the construction of high-resolution molecular atlases of brain cell populations and enhancing our understanding of neuronal diversity and the molecular determinants of functional specialization (Fig. 2) [19, 30, 31]. Each modality occupies a distinct position within a trade-off space defined by spatial resolution, molecular depth, cellular coverage, tissue context, and computational scalability. Therefore, interpreting large-scale brain multi-omics atlases requires explicit consideration of what each technology captures, what it loses, and what biological claims it can support (Table 1).

**Table 1:** Multi-omics mapping technologies for brain research.

Technology	Platforms	Advantages in brain	Disadvantages	Biological insights
Single-cell/nuclei transcriptomics	10x Genomics [62]; DNBelab C4 [63]; Stereo-cell [64]; SMART-seq2 [65]; CEL-seq [66]; Drop-seq [67]	Unbiased single-cell transcriptome profiling	Spatial loss; dissociation bias; dropout noise	Cellular heterogeneity and state transitions
Single-cell/nuclei epigenomics	10x Chromium ATAC [68]; sci-ATAC-seq [69]; CH-ATAC-seq [70]	Cell-resolved regulatory landscape mapping	Sparse signal; indirect expression linkage	Regulatory programs and noncoding variation
Single-cell proteomics	CITE-seq [37]; Chip-Tip [36]	Direct measurement of protein phenotype	Limited coverage; antibody panel constraints	Functional states and signaling activity
Single-cell metabolomics	scMEP [71]	Single-cell metabolic-state inference	Limited analyte coverage; indirect flux readout	Metabolic heterogeneity and pathway rewiring
Single-cell genomics	MDA [72]; MALBAC [73]	Single-cell resolution of somatic mutation and copy-number variation	Amplification bias; low throughput	Clonal structure and genome instability
Single-cell multi-omics	SIDR [43]; scCAT-seq [74]; SPLIT-seq [75]; SHARE-seq [40]	Direct genotype–epigenome–transcriptome coupling within single cells	Reduced depth; complex integration	Genotype-regulation-phenotype coupling
Spatial transcriptomics	Stereo-seq [48]; Stereo-seq V2 [49]; 10x Visium [76]; MERFISH [77]; Slide-seq [78]; STARmap [79]	Transcriptomics with preserved tissue context	Variable resolution; mixed-cell signals	Spatial niches and regional programs
Spatial epigenomics	Spatial-ATAC-seq [61]; Spatial-CUT&Tag [80]	Spatial mapping of chromatin accessibility and histone modification states in intact tissue	Low sensitivity; limited standardization	Layer-specific regulatory programs and spatially restricted chromatin remodeling
Spatial proteomics	Stereo-CITE-seq [81]; CODEX [53]; MIBI [55]	*In situ* quantification of protein states	Panel-limited; validation-intensive	Tissue neighborhoods and signaling niches
Spatial metabolomics	SEAM [56]; scSpaMet [82]	Spatial profiling of metabolites and lipids	Incomplete identification; limited cell attribution	Metabolic zonation and biochemical gradients
Spatial multi-omics	SM-Omics [58]; DBiT-seq [59]; Spatial CITE-seq [83]; Spatial ATAC-RNA-seq [80]	Simultaneous multi-modal profiling with spatial tissue context preserved	Lower depth; demanding integration	Coordinated molecular programs in context

Within the single-cell omics technology framework, single-cell RNA sequencing (scRNA-seq) has become a fundamental tool for identifying neuronal and glial cell types. Widespread use of commercial platforms (e.g., 10x Genomics, DNBelab C4, Stereo-cell, SMART-seq) has enabled researchers to establish detailed cellular taxonomies for brain regions across multiple species [19, 30–33]. However, tissue dissociation introduces transcriptional stress artifacts and selectively depletes fragile cell populations such as large projection neurons, while spatial context and cell–cell contact information are irretrievably lost. Concurrently, single-cell chromatin accessibility assays (scATAC-seq/snATAC-seq) provide crucial insights into the epigenetic mechanisms governing brain cell development and differentiation by revealing chromatin openness that dictates cell fate [34, 35]. The primary limitation remains signal sparsity, because each nucleus yields only a limited number of accessible chromatin fragments, leading to dropout and reduced sensitivity for distal regulatory elements. Single-cell proteomic technologies (e.g., CITE-seq, Chip-Tip) facilitate the detection of cell surface proteins, aiding in the fine subtyping and functional state analysis of immune cells (like microglia) in the central nervous system [36, 37]. Nevertheless, CITE-seq is constrained by antibody panel size, and neither approach captures the spatial tissue context in which protein states are regulated. Single-cell metabolomics technologies, primarily based on mass spectrometry approaches such as scMEP (single-cell metabolic profiling by epitope-based), enable the characterization of hundreds of metabolites in individual neurons and glial cells, revealing metabolic heterogeneity between cell types and functional states [38, 39]. These technologies have proven particularly valuable for neuroscience research by detecting neurotransmitters, amino acids, lipids, and other small molecules that reflect the biochemical state of individual brain cells, providing insights into neuronal activity, cellular plasticity, and metabolic dysregulation in brain disorders. Furthermore, joint single-cell multi-omics technologies (e.g., SIDR, SPLIT-seq, SHARE-seq), capable of capturing multiple molecular layers from the same cell, provide direct evidence for constructing complete gene regulatory networks, significantly advancing our comprehension of the mechanisms determining brain cell identity [40–44]. Single-cell genomics approaches, utilizing whole genome amplification and high-throughput sequencing, have revealed extensive somatic mutations in individual neurons, including single-nucleotide variants, copy number variations, and retrotransposon insertions [45]. These approaches have revealed that individual neurons can harbor hundreds to thousands of somatic mutations that accumulate during development and aging. Such mutations can serve as endogenous lineage markers and provide insights into brain development, neuronal diversity, and neurological disorders, including epilepsy, autism spectrum disorder (ASD), and focal cortical dysplasia [45, 46].

However, conventional single-cell techniques inevitably lose the native spatial context of cells during tissue dissociation [47]. Given that the brain is a highly structured organ whose function strictly depends on precise spatiotemporal architecture, this limitation posed a critical challenge, spurring the rapid advancement of spatial omics technologies. High-resolution spatial transcriptomic techniques like Stereo-seq [48] and its enhanced version Stereo-seq V2 [49], with their subcellular resolution and whole-transcriptome coverage, allow molecular expression data to be precisely anchored to their original tissue locations. This enables the direct “observation” of distribution patterns for specific cell types and genes across different brain cortices, nuclei, and even fine laminar structures. Similarly, other technologies like 10x Visium, MERFISH, Slide-seq, and STARmap, each with unique strengths in resolution, throughput, and multi-gene detection capability, complement each other and collectively propel the application of spatial transcriptomics in brain research [1, 30, 50–52]. Array-based spatial transcriptomic methods can provide broad or whole-transcriptome coverage, but many sacrifice per-spot sensitivity or cellular resolution, especially when each capture area contains multiple cells. By contrast, imaging-based methods can achieve single-cell or subcellular resolution, but they are usually restricted to predefined gene panels, limiting unbiased transcriptome-wide discovery.

Currently, spatial multi-omics technologies are driving brain science from a singular transcriptomic dimension toward a new phase integrating spatial information for proteins, metabolites, and chromatin. Spatial proteomic technologies (e.g., Stereo-CITE, CODEX, MIBI) enable the simultaneous *in situ* detection of dozens of proteins, offering novel perspectives for studying brain cellular spatial organization and cell–cell interactions [53–55]. Spatial metabolomics technologies (e.g., SEAM) can reveal the distribution of small molecule metabolites in brain tissue, linking metabolic states to functional brain regions [56]. Truly revolutionary joint spatial multi-omics technologies (e.g., DBiT-seq, Spatial-ATAC-RNA-seq, Spatial CITE-seq, SM-Omics) go a step further, allowing researchers to concurrently obtain transcriptomic and epigenomic information from the same tissue section, providing possibilities for understanding the epigenetic regulation of gene expression within the native microenvironment [57–61]. Current limitations include pixel- or spot-based resolution rather than robust single-cell segmentation in many platforms, as well as lower per-modality depth compared with unimodal assays. As a result, many brain applications of joint spatial multi-omics remain limited in scale or at the proof of concept stage.

The integration of these multi-omics technologies has established a framework for multi-dimensional, high-resolution brain cell atlas construction. These approaches enable systematic investigation of the brain’s cellular architecture, molecular regulatory networks, and functional mechanisms, and provide methodological tools for examining brain dynamics across development, aging, and disease states. Continued refinements in resolution, throughput, and multi-modal integration capabilities are advancing efforts toward comprehensive characterization of brain complexity.

## Multi-omics brain atlas: a panoramic view from health to disease

**Figure 3:**
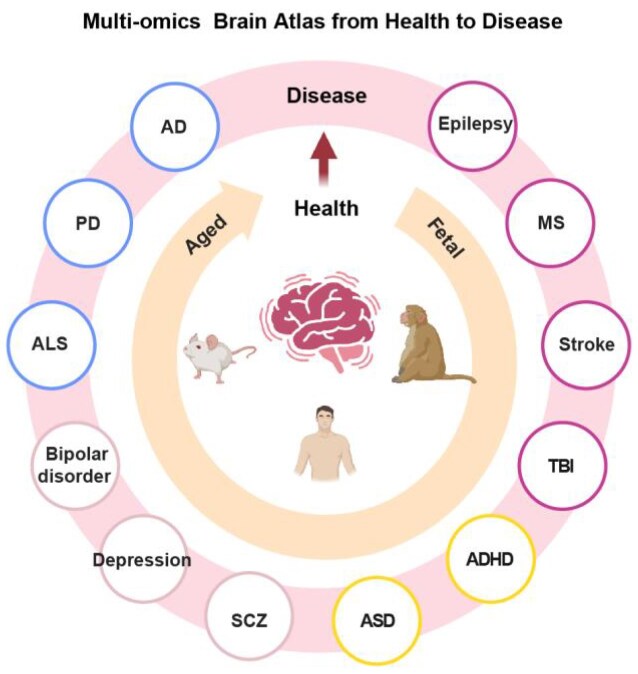
The schematic of multi-omics brain atlas spanning from healthy states to diverse pathological conditions. The central core represents the healthy brain atlas constructed from multi-omics profiling across model organisms (rodents, non-human primates, and humans), serving as the reference foundation. The inner ring depicts temporal dimensions spanning fetal development to aged stages, highlighting dynamic changes throughout the lifespan. The outer ring encompasses brain disorders investigated through atlas-based approaches: neurodegenerative diseases (AD, PD, ALS), psychiatric disorders (depression, SCZ, bipolar disorder), neurodevelopmental disorders (ASD, ADHD), and other neurological conditions (epilepsy, MS, stroke, TBI). The shared biological themes emerging across these disease atlases include selective vulnerability of defined neuronal populations, activation and state transitions of glial cells, synaptic and circuit dysfunction, and dysregulation of developmental or maturation-related programs.

The construction of comprehensive brain atlases represents a critical bridge connecting multi-omics technologies to clinical applications in precision neurology. These atlases provide systematic reference frameworks that span the entire spectrum from healthy brain states to diverse pathological conditions, enabling researchers to identify disease-specific cellular and molecular alterations through comparative analysis [84]. This integrated approach encompasses multiple dimensions: it leverages multi-omics profiling across various model organisms to establish robust baseline references [85], tracks dynamic changes throughout developmental and aging trajectories [86], and systematically investigates a broad range of brain disorders, including neurodegenerative diseases, psychiatric conditions, and neurodevelopmental disorders [87] (Fig. 3). This section examines how multi-omics brain atlases are being systematically constructed for both healthy and diseased states, demonstrating their transformative impact on understanding brain function and dysfunction.

## Multi-omics cellular mapping of the healthy human brain

The human brain represents a biological system of exceptional structural and functional complexity. Constructing a comprehensive cellular atlas of human brain is pivotal to understanding neural function, developmental processes, aging-related changes, and the pathophysiology of neurological disorders [88]. Recent advances in single-cell sequencing and spatial omics technologies have enabled a transition from region-based neuroanatomical descriptions to cellular-resolution characterization [89, 90]. This shift has facilitated the systematic cataloging of cell types and their molecular profiles, enabling the construction of multi-scale, high-resolution atlases of the healthy human brain.

Large-scale brain cell atlas initiatives, exemplified by the Human Brain Cell Atlas (HBCA), are revolutionizing our understanding of cerebral cellular architecture [91]. By integrating single-cell multi-omics data across regions and development, these projects have unveiled an unprecedented degree of cellular diversity and region-specific specialization [85]. Spatially resolved transcriptomics and computational methods have been pivotal in mapping this complexity, leading to a refined neuronal taxonomy and the discovery of extensive functional heterogeneity among glial cells [89, 92]. Furthermore, cellular-resolution maps of key structures like the hippocampus and cerebellum are directly elucidating the cellular substrates of learning, memory, and motor coordination, moving beyond anatomical description to mechanistic insight [93].

Extending the perspective to developmental timelines, brain atlas research has further uncovered the dynamic blueprint of brain construction. Spatiotemporal atlas studies of the fetal brain have meticulously delineated the molecular programs governing neurogenesis, neuronal migration, and synaptogenesis. Multi-omics analyses of critical developmental windows have provided new insights into the regulatory mechanisms driving the brain’s rapid dynamic changes. The precise spatiotemporal regulation of gene expression during development not only shapes a healthy brain but also offers crucial clues for understanding interindividual differences in neuroplasticity and susceptibility to brain disorders [86, 94].

In summary, by spanning macroscopic regions to microscopic cells and transitioning from static classifications to dynamic development, integrated multi-omics brain atlases provide a comprehensive framework for understanding the brain in health. This continuously expanding and refining blueprint will serve as an increasingly powerful reference framework for decoding brain organization, while its interpretation in disease contexts requires careful consideration of cell-type uncertainty, region-specific taxonomy, dynamic cell states, and disease-induced deviations from healthy references.

To provide a structured overview of currently available reference resources, representative healthy or reference brain atlases generated from single-cell, single-nucleus, spatial, and multi-omics studies are summarized [63, 89, 93, 95–110]. These resources are compared in terms of species, brain regions, developmental stages, omics modalities, spatial information, data accessibility, major strengths, limitations, and their relevance to the multi-omics construction of next-generation brain atlases in neurology (Table S1). This comparison highlights that healthy reference atlases provide essential baseline cell taxonomies and molecular frameworks, but their use for disease interpretation requires careful consideration of sampling coverage, donor diversity, spatial resolution, modality depth, and compatibility across platforms.

## Conceptual and computational caveats in brain cell atlas interpretation

Although healthy brain cell atlases provide indispensable reference frameworks for interpreting cellular diversity, their use in disease mechanism inference and precision neurology requires careful consideration of several conceptual and computational limitations.

First, cell type definitions are not fully stable across studies [111–113]. In single-cell and spatial atlas projects, cell taxonomies can be influenced by brain region selection, developmental stage, donor composition, species, sequencing platform, tissue dissociation or nuclei isolation protocol, sequencing depth, batch correction strategy, clustering resolution, and annotation algorithm [114–116]. As a result, the same biological population may be described as a class, subclass, subtype, cluster, or state in different studies. Therefore, cell labels in brain atlases should be interpreted as hierarchical and context-dependent annotations [93, 113] rather than absolute biological entities. Whenever possible, cell type assignment should be supported by multiple layers of evidence, including transcriptomic signatures, chromatin accessibility, spatial localization, morphology, connectivity, physiology, and functional validation [99, 117].

Second, region-specific and whole-brain taxonomies serve different purposes and should not be treated as interchangeable [99, 118]. Whole-brain taxonomies provide a harmonized framework for cross-region comparison and large-scale integration, whereas region-specific atlases can resolve local cellular specialization, laminar organization, developmental trajectories, and microenvironmental niches with higher precision. However, whole-brain atlases may merge regionally specialized subtypes, while region-specific atlases may over-split local populations that are difficult to align across brain regions. Thus, findings derived from cortical, hippocampal, cerebellar, or fetal brain atlases should not be generalized to all brain regions or all neurological diseases without considering region, age, developmental stage, and disease context.

Third, cell type and cell state should be clearly distinguished [111, 119]. A cell type generally refers to a relatively stable cellular identity shaped by developmental lineage, gene regulatory programs, molecular phenotype, morphology, connectivity, and function. In contrast, a cell state refers to a dynamic and potentially reversible condition induced by aging, neuronal activity, inflammation, injury, medication, technical stress, or disease. For example, disease-associated microglia, reactive astrocytes, stressed neurons, or inflammatory endothelial cells may represent context-dependent states within broader cell types rather than entirely new cell types. This distinction is essential when interpreting disease-associated cellular changes, because changes in cell state may reflect pathological response, compensatory adaptation, technical artifact, or secondary effects rather than primary disease drivers.

Finally, mapping diseased cells onto healthy reference atlases introduces substantial uncertainty [115, 120]. Diseased tissues may contain transcriptionally shifted cells, transitional phenotypes, infiltrating immune cells, degenerating cells, reactive glia, or rare pathological cell states that are absent or underrepresented in healthy references [120]. Computational label transfer methods may force such cells into the nearest healthy category, thereby obscuring novel disease-associated states or exaggerating apparent cell-type specificity [121]. To reduce this risk, atlas-based disease interpretation should incorporate label confidence scores, out-of-distribution detection, matched healthy and disease controls, disease-specific reference atlases, cross-atlas validation, and spatial or histopathological confirmation [122]. Accordingly, mapping diseased cells to healthy brain cell atlases should be regarded as a hypothesis-generating strategy rather than definitive mechanistic proof. These caveats are particularly important for precision neurology, where disease subtype stratification, biomarker discovery, and therapeutic target prioritization depend on robust and reproducible cell identity assignments [123].

## Multi-omics cellular mapping of brain disorders

Building on the conceptual and computational caveats discussed above, disease-oriented single-cell and spatial multi-omics studies should be interpreted not as a simple one-to-one extension of healthy reference atlases, but as a framework for identifying reproducible cell-type- and cell-state-specific alterations in pathological contexts. When combined with disease-matched controls, spatial localization, histopathological annotation, and orthogonal multi-omic validation, atlas-based analyses can help distinguish stable cell identities from dynamic disease-associated states, transitional phenotypes, infiltrating or degenerating cell populations, and spatially restricted pathological niches. With these considerations in mind, a major goal of disease-oriented brain cell atlas research is to elucidate the cellular and molecular mechanisms underlying neurological and psychiatric disorders [12]. Through the synergistic integration of multidisciplinary tools, including single-cell multi-omics, spatial omics, neuroimaging, and computational modeling, researchers are increasingly able to deconstruct disease-relevant neural architecture across multiple biological scales, ranging from molecular pathways and synaptic alterations to cell–cell interactions, regional circuits, and large-scale functional networks [91, 93]. Such knowledge provides a mechanistic basis for identifying disease-vulnerable cell populations, discovering biomarkers, prioritizing therapeutic targets, and developing targeted interventions for disorders ranging from AD to psychiatric conditions [84].

Single-cell multi-omics studies have profoundly advanced our understanding of neurological diseases, especially AD. By simultaneously analyzing multiple layers of biological information, including gene expression and epigenetic modifications, researchers have uncovered intricate molecular alterations within specific brain cell types that fundamentally reshape our understanding of AD progression [84, 124–126], offering a novel analytical dimension for understanding disease mechanisms and identifying new therapeutic targets. Multi-omics studies have delineated amyloid-β plaque-associated cellular responses and transitions of microglia and other glial populations toward disease-associated states [127–129]. In the effort of systematically research using multi-omics, AD is increasingly viewed as a disorder of cellular networks, where the interplay between proteopathy and glial responses determines outcome, highlighting the therapeutic promise of precisely modulating microglial phenotypes alongside targeting Tau propagation [130, 131]. Beyond AD, single-cell multi-omics studies have expanded to elucidate a broad spectrum of neurological disorders, each with distinct pathological features. For instance, in PD, multi-omics analyses have uncovered the molecular underpinnings of dopaminergic neuron loss in the substantia nigra and the cellular responses surrounding Lewy body formation, revealing dysregulated pathways in protein aggregation and neuroinflammation. In amyotrophic lateral sclerosis (ALS), integrated omics approaches have delineated the degenerative processes in motor neurons and altered neuron-glia interactions, highlighting key genes involved in excitotoxicity and immune activation [132–136]. For ASD, these studies have exposed cortical developmental anomalies and synaptic connectivity alterations, implicating epigenetic modifications and neural circuit dysfunctions [137]. Similarly, ADHD investigations have mapped cell-type-specific changes in relevant brain regions, uncovering expression variations in genes related to neurodevelopment and neurotransmission [138]. In multiple sclerosis (MS), multi-omics data have detailed the immune cell infiltration patterns in demyelinating lesions, providing insights into autoimmune mechanisms and potential repair processes [139].

Moving to acute brain injuries, multi-omics approaches have been instrumental in decoding the dynamic cellular responses. In stroke, single-cell analyses of the ischemic penumbra have characterized the complex cell reactions and neurorepair mechanisms, including angiogenesis, glial scar formation, and axonal regeneration [15]. For traumatic brain injury (TBI), omics technologies have captured the spatiotemporal dynamics of injury cascades, such as oxidative stress, inflammation, and cell death pathways, offering a holistic view of recovery and degeneration [140].

In the realm of psychiatric disorders, multi-omics studies have begun to unravel the molecular basis of complex conditions. Schizophrenia research has focused on dopaminergic system dysregulation and prefrontal cortical cell alterations, with omics data identifying aberrant gene networks in synaptic function and immune signaling. Depression studies have emphasized impairments in hippocampal neurogenesis and neuroinflammatory features, linking transcriptomic and epigenetic changes to stress responses and treatment outcomes. For bipolar disorder, multi-omics analyses have revealed molecular abnormalities in emotion-regulation brain regions, including alterations in circadian rhythm genes and neurotransmitter systems, paving the way for personalized therapeutic strategies.

We also summarized disease-focused brain atlas resources, with representative disease datasets compared in terms of disease category, tissue source, brain region, omics modality, spatial information, data availability, major biological insights, strengths, and limitations [46, 87, 129, 139, 141–157] (Table S1). This addition helps distinguish broadly reusable disease resources from smaller disease-specific studies and highlights how disease atlases contribute to precision neurology.

## Shared and replicated cellular programs across brain disorders

Across disease-focused single-cell and spatial multi-omics studies, several recurrent biological themes have emerged across independent cohorts, brain regions, and disease categories. First, many disorders show selective cellular vulnerability, in which specific neuronal populations are disproportionately affected relative to neighboring cell types [87, 158, 159]. Examples include dopaminergic neurons in PD, motor neurons in ALS, cortical projection neurons and synapse-associated neuronal populations in AD and psychiatric disorders, and developmentally regulated cortical cell populations in neurodevelopmental disorders. Although the exact vulnerable subtype may differ across anatomical regions and disease stages, the recurring observation is that disease risk and pathology are not uniformly distributed across all brain cells but are concentrated in defined cellular compartments.

Second, glial activation and glial state transitions represent a shared feature across neurodegenerative, inflammatory, and injury-related disorders [119, 160, 161]. Microglia frequently adopt disease-associated, inflammatory, phagocytic, or interferon-responsive states, whereas astrocytes may acquire reactive programs associated with cytokine signaling, metabolic remodeling, and synaptic support. Oligodendrocyte-lineage cells and vascular-associated cells are also increasingly recognized as contributors to disease progression, particularly in demyelinating, ischemic, and degenerative contexts. These glial programs are repeatedly detected across studies, but their precise marker genes, directionality, and functional consequences can vary according to disease stage, tissue region, postmortem interval, and analytical strategy.

Third, developmental and maturation-related dysregulation is a common theme in neurodevelopmental and psychiatric disorders, and may also be partially reactivated in adult-onset neurodegenerative disease as part of stress or repair responses [148, 162, 163]. Altered chromatin accessibility, disrupted synaptic gene programs, impaired neuronal maturation, and perturbed cell–cell communication have been observed across ASD, schizophrenia, intellectual disability, and other developmental conditions. Together, these findings suggest that brain disease atlases should be interpreted not only as catalogs of disease-specific alterations but also as maps of shared cellular vulnerability, glial response, and developmental or maturation-state imbalance.

Importantly, the degree of replication differs by biological resolution. Broad cell-class-level changes and pathway-level signatures, such as neuronal vulnerability, immune activation, synaptic dysfunction, and developmental dysregulation, are generally more reproducible across studies than individual marker genes or highly resolved disease-associated cell states [152, 160, 164]. Variation in cohort composition, brain region, disease stage, sequencing platform, nuclei versus whole-cell preparation, and computational annotation can all influence the apparent disease signatures. Therefore, replicated disease features should ideally be evaluated across multiple datasets and, where possible, supported by spatial localization, epigenomic evidence, proteomic validation, or experimental perturbation.

Collectively, these multi-omics investigations across diverse brain disorders not only deepen our understanding of disease-specific mechanisms but also foster the development of precision medicine approaches. By integrating data from genomics, transcriptomics, and epigenomics at single-cell resolution, we are building a comprehensive atlas of brain pathologies that bridges molecular insights with clinical applications, ultimately advancing our mission to mitigate the global burden of neurological and psychiatric illnesses.

## Cross-species brain atlas: an evolutionary biology perspective

A critical dimension underlying the validity of these disease atlases, however, is the degree to which findings derived from model organisms can be meaningfully extrapolated to human pathophysiology. Cross-species comparative atlas construction directly addresses this question, providing an evolutionary framework for distinguishing conserved disease mechanisms from species-specific biological features, and for rationally calibrating the translational relevance of non-human model systems. The Brain Initiative Cell Census Network (BICCN) has provided a comprehensive framework for such analyses by generating cellular atlases across human, non-human primates (rhesus macaque and marmoset), and mouse brains [91, 100]. At the foundational level, comparative multi-omics studies reveal striking evolutionary conservation of basic cellular architecture. Most fundamental cell types, including major neuronal and glial cell classes, are highly conserved across mammalian species [165], and certain cell-type-specific molecular markers and signaling pathways exhibit remarkable cross-species consistency [91, 166, 167]. This conservation provides reliable anchor points for translational research and validates the use of animal models for investigating core neurobiological mechanisms. However, layered upon this conserved foundation are significant species-specific elaborations that distinguish the human brain. The most prominent anatomical feature is the substantial expansion of the human neocortex, particularly in prefrontal and temporal association cortices, which correlates with enhanced cognitive capabilities. At the cellular level, single-cell transcriptomic analyses have identified specifically expanded neuronal subtypes in the human cortex, with these cells expressing human-specific genes potentially contributing to higher-order cognitive functions [168, 169]. Notably, the human cortex displays elevated proportions of upper-layer projection neurons and more complex interneuron subtypes [170]. Beyond changes in cellular composition, species-specific differences manifest in cellular proportions, subtype classifications, and gene expression levels [171]. Complementary epigenomic studies have illuminated the regulatory mechanisms underlying these evolutionary innovations. Comparative analyses have identified human-specific regulatory elements enriched near genes related to neural development and synaptic function [172]. These findings underscore the pivotal role of gene regulatory evolution, as opposed to coding sequence changes alone, in driving the unique trajectories of human brain evolution [173]. These evolutionary divergences carry important implications for translational neuroscience. Cross-species comparisons have revealed that genes associated with neurodegenerative diseases, such as AD, are not always expressed in equivalent cell types between humans and mice [158, 174]. This cellular-level species specificity necessitates cautious interpretation when extrapolating findings from animal models to human pathophysiology, highlighting the importance of integrating human-derived data into preclinical research paradigms.

To clarify the translational value and limitations of model organisms, representative single-species and multi-species atlas resources are compared [89, 95, 99, 171, 175–177]. This table indicates whether each resource supports direct cross-species alignment, whether data are publicly available, and how each atlas contributes to conserved mechanism discovery, model selection, and human disease interpretation (Table S1).

## Robustness and limitations of cross-species cell-type alignment

The robustness of cross-species alignment is highly dependent on cellular resolution and molecular feature type. At the broadest level, major brain cell classes, including excitatory neurons, inhibitory neurons, astrocytes, oligodendrocytes, oligodendrocyte precursor cells, microglia, endothelial cells, and mural cells, can generally be aligned across human, non-human primates, and mouse using conserved marker genes and shared transcriptional programs [99, 100, 165]. These broad correspondences provide reliable anchors for comparative atlas construction and support the use of model organisms to investigate fundamental neurobiological mechanisms.

However, alignment becomes progressively less robust at finer cellular resolution [100, 170]. Neuronal subclasses, cortical layer-specific projection neuron populations, interneuron subtypes, transient developmental populations, and disease-associated glial states may show partial, context-dependent, or species-specific correspondence. Several factors contribute to this limitation, including incomplete one-to-one orthology of genes, differences in cell-type proportions, divergent developmental timing, region-specific expansion of primate and human cortical populations, and species-specific regulatory elements. In addition, disease-associated cellular states may reflect not only intrinsic cell identity but also environmental context, immune activation, aging, and pathology burden, making their cross-species matching more challenging than alignment of stable homeostatic cell types.

Feature conservation also varies across molecular layers. Canonical cell identity markers and core signaling pathways tend to be more conserved, whereas enhancer usage, chromatin accessibility, non-coding regulatory elements, gene expression magnitude, cell-type proportions, and disease-induced state transitions are often more species-specific [24, 178]. Therefore, cross-species comparisons are most robust when they integrate multiple levels of evidence, including transcriptomic similarity, epigenomic regulatory conservation, spatial localization, developmental trajectory, and functional validation [93]. In this framework, mouse models remain powerful for mechanistic perturbation of conserved pathways, non-human primates provide closer models for primate-specific cortical architecture and higher-order cell types, and human tissues or organoid systems are essential for validating human-specific regulatory and disease-associated features (Table 2).

**Table 2:** Replicated and shared cellular themes across brain disorders and cross-species conservation.

Biological theme	Representative references	Representative disorders	Main cell types/states	Replication across studies	Cross-species conservation	Limitations
Selective cellular vulnerability	Mathys et al. [158]; Kamath et al. [87]; Limone et al. [186]; Li et al. [163]	AD, PD, ALS, SCZ, ASD	Dopaminergic neurons, motor neurons, cortical projection neurons, interneuron subtypes	Recurrent at cell-class or pathway level	Broad neuronal classes conserved; fine subtypes less robust	Region, disease stage, and annotation differences
Glial activation	Keren-Shaul et al. [119]; Deczkowska et al. [187]; Olah et al. [164]; Garza et al. [161]; Bormann et al. [160]	AD, MS, ALS, stroke, TBI, depression	Microglia, astrocytes, oligodendrocyte-lineage cells	Frequently observed across datasets	Core immune/stress pathways partly conserved	Disease-associated glial states may be species- and context-dependent
Synaptic and circuit dysfunction	Gandal et al. [148]; Mathys et al. [158]; Nagy et al. [188]	AD, SCZ, ASD, depression	Excitatory/inhibitory neurons, synaptic compartments	Recurrent across neurodegenerative and psychiatric disorders	Many synaptic pathways conserved	Human cortical circuitry and cell proportions differ from mouse
Developmental or maturation dysregulation	Gandal et al. [148]; Jin et al. [162]; Nagy et al. [188]	ASD, ADHD, SCZ, intellectual disability	Progenitors, immature neurons, cortical projection neurons	Strong theme in developmental and psychiatric disorders	Developmental programs partly conserved	Timing and cortical expansion differ across species
Vascular and immune microenvironment	Yang et al. [84]; Wälchli et al. [94]; Lerma-Martin et al. [152]; Garza et al. [161]	MS, stroke, TBI, AD	Endothelial cells, pericytes, immune cells, microglia	Increasingly replicated in spatial and single-cell studies	Broad vascular cell classes conserved	Injury and inflammation responses vary by model

Mice represent the most commonly used model organism in neuroscience research, with their genomic manipulability and relatively short lifespan making them ideal tools for studying brain development and disease mechanisms [179]. However, cross-species atlas comparisons have revealed important limitations of mouse models [180]. The human brain exhibits substantial differences from mice in size, cortical parcellation, and cellular composition [171], with certain human-specific cell types and gene expression patterns being absent or expressed at very low levels in mice [170]. These differences represent one potential contributor to the frequent failure of therapeutic strategies [181]. In addition, non-human primates, due to their closer phylogenetic relationship to humans, more closely resemble humans in brain structure and cognitive function, and are therefore considered more ideal models for translational research [182, 183]. Single-cell atlas comparisons demonstrate that humans and non-human primates exhibit high similarity in cell type composition and gene expression patterns, particularly in brain regions associated with higher-order cognition [184]. Nevertheless, even in non-human primates, significant differences from humans persist, including the absence of human-specific genes and variations in certain cell type proportions [169]. Furthermore, non-human primate research faces ethical, financial, and technical challenges that limit its widespread application.

To overcome the limitations of single-species models, multi-species integrative research strategies are emerging. By conducting mechanistic studies and genetic manipulation experiments in mouse models, validating key findings in non-human primate models [185], and integrating human brain tissue and organoid studies [91], researchers can establish a more complete and reliable framework for disease understanding and therapeutic target validation. Cross-species cellular atlases provide an essential reference framework for such integrative research, helping researchers identify conserved and species-specific mechanisms and rationally select and interpret animal model research findings.

### AI empowerment: large language foundation models revolutionizing brain multi-omics

The unprecedented complexity of the brain has long resisted systematic computational interpretation. This complexity arises from hundreds of molecularly distinct cell types, intricate spatial organization, and disease-associated genetic variations distributed across vast non-coding regulatory landscapes [189]. However, the emergence of large language foundation models (FMs) trained on biological sequences offers a transformative opportunity. By learning the statistical grammar of DNA, transcriptomes, and proteins from tens of millions of sequences, these models derive representations that generalize across tasks, datasets, and species in ways that task-specific models cannot. This section surveys representative FMs across 4 domains, namely genomics, single-cell transcriptomics, spatial omics, and clinical medicine, emphasizing their architecture, scale, and direct relevance to brain science [190] (Table 3).

**Table 3:** AI foundation models applied in brain multi-omics research.

Model	Author and time	Task	Brain science focus
DNABERT-2	Zhou et al. [191]	DNA	Predicts regulatory elements and noncoding variants across species
HyenaDNA	Nguyen et al. [192]	DNA	Models long-range genomic interactions at single-nucleotide resolution
Genos	Lin et al. [193]	DNA	Analyze the human genomic data of a large population
Evo 2	Brixi et al. [194]	DNA	Predicts pathogenic noncoding variants across eukaryotic genomes
AlphaGenome	Avsec et al. [195]	DNA	Predicts gene expression, chromatin accessibility, and regulatory variant effects from DNA sequence
AlphaFold2	Jumper et al. [196]	Protein	Predicts protein 3D structures from sequences; models Aβ42 aggregation in Alzheimer’s disease
AlphaFold3	Abramson et al. [197]	Protein	Predicts biomolecular complex structures; characterizes structural effects of AD-associated missense variants
ESM-2	Frank et al. [198]	Protein	Predicts protein structure and amyloid aggregation propensity of tau, APP, and α-synuclein
AlphaMissense	Cheng et al. [199]	Protein	Classifies missense variant pathogenicity across neurological disease genes (LRRK2, SNCA, APP, PSEN1)
AlphaPeptDeep	Zeng et al. [200]	Protein	Predicts peptide RT, CCS, and MS2 intensities for PTM-rich brain proteome DIA analysis
scGPT	Cui et al. [201]	scRNA	Annotates cell types and integrates batches from single-cell brain transcriptomics data
Geneformer	Theodoris et al. [202]	scRNA	Predicts gene dosage sensitivity and network biology in fetal brain cells
scFoundation	Hao et al. [203]	scRNA	Predicts cell-type-specific drug responses across CNS cell populations
UCE	Rosen et al. [204]	scRNA	Annotates cell types across species without fine-tuning using universal cell embeddings
GeneCompass	Yang et al. [205]	scRNA	Annotates mouse brain cell types with knowledge-guided gene regulatory integration
CellFM	Zeng et al. [206]	scRNA	Annotates rare brain cell populations including disease-associated microglia and interneuron subtypes
CAPTAIN	Ji et al. [207]	scRNA	Models joint RNA–protein representations and intercellular dynamics in neural microenvironments
Nicheformer	Tejada-Lapuerta et al. [208]	Spatial	Characterizes tissue niches and brain region identity from spatial transcriptomics data
Novae	Blampey et al. [209]	Spatial	Identifies spatial domains and parcellates brain regions across unseen gene panels
OmiCLIP	Chen et al. [210]	Spatial	Predicts spatial transcriptomics from H&E histology images for brain cell-type decomposition
scGPT-spatial	Wang et al. [211]	Spatial	Decodes, imputes, and deconvolves spatial gene expression across brain tissue sections
OmniCell	Pang et al. [212]	Spatial	Models intra- and inter-cellular spatial dependencies in aging mouse brain atlas
Med-PaLM 2 & Med-PaLM M	Singhal et al. [213]	Medical LLM	Answers medical questions and analyzes multimodal neurological cases at expert level
GatorTron	Yang et al. [214]	Medical LLM	Extracts psychiatric and neurological information from unstructured clinical notes
GatorTronGPT	Peng et al. [215]	Medical LLM	Generates synthetic clinical text and supports psychiatric decision-making
Meditron	Chen et al. [216]	Medical LLM	Predicts ICU mortality for patients with mental disorders from clinical notes

## Genomic FMs

More than 90% of neurological disease-associated variants identified by GWAS reside in non-coding regulatory regions, yet the functional interpretation of these variants has remained a bottleneck. Genomic foundation models (gFMs) address this by learning the regulatory grammar encoded in DNA sequence, evolving from transformer-based designs toward hybrid architectures capable of processing megabase-scale contexts at single-nucleotide resolution.

DNABERT-2 [191] introduced byte pair encoding to replace the *k*-mer tokenization of its predecessor, dramatically reducing computational cost while achieving competitive performance across genomic classification tasks. Its ability to generalize zero-shot to sequences far longer than those seen during training makes it particularly applicable to the large, complex regulatory domains that govern brain-specific gene expression programs. While DNABERT-2 extended breadth of genomic representation, HyenaDNA extended depth of context. By replacing the transformer’s attention mechanism with Hyena operators, HyenaDNA achieves context lengths of up to 1 million base pairs with sub-quadratic scaling [192]. This represents a decisive advance for neuroscience, as neuronal enhancers and silencers often reside hundreds of kilobases from their target genes. This capacity to model distal regulatory interactions is essential for decoding the cell-type-specific gene programs of neurons and glia whose dysregulation underlies much of neurological disease. Building on this foundation of scale and context, Genos pushes genomic modeling to a new tier of population diversity and clinical resolution. As one of the largest gFMs to date, Genos employs a mixture-of-experts transformer with 10.27 billion parameters and 1-megabase context, trained on 636 telomere-to-telomere human genome assemblies spanning diverse global populations [193]. The model achieves 93% area under the curve (AUC) on pathogenicity prediction and has demonstrated direct neurological application in variant effect scoring for LRRK2, the gene most commonly mutated in familial PD. This success exemplifies how population-scale genomic modeling can be grounded in cell-type-specific neuropathology.

The Arc Institute’s Evo series extends this logic across the full diversity of life. The first-generation Evo employs a 7-billion-parameter StripedHyena architecture trained on the OpenGenome dataset spanning bacteria, archaea, and bacteriophages, enabling zero-shot gene essentiality prediction and de novo multi-gene circuit generation at single-nucleotide resolution [217]. Its successor, Evo 2, substantially expands scale and biological scope: 7B and 40B parameter variants trained on approximately 9.3 trillion DNA tokens from all domains of life, with 1 million token context windows and a hybrid Transformer–StripedHyena architecture [194]. Critically, Evo 2 extends to eukaryotic genomes, including human, enabling prediction of pathogenic noncoding mutations and clinically relevant variant effects. For brain research, this cross-domain generalization is particularly significant: regulatory variants driving neurological disease often reside in non-coding sequence elements whose evolutionary conservation and functional logic become interpretable only in the context of deep evolutionary diversity. Whereas the preceding models focus on variant interpretation within linear sequence context, AlphaGenome, developed by Google DeepMind, integrates the outputs of genomic regulation into a unified multi-modal prediction framework. Accepting up to 1 megabase of DNA, AlphaGenome simultaneously predicts across 8 functional genomic modalities: gene expression, transcription initiation, chromatin accessibility, histone modifications, transcription factor binding, chromatin contact maps, splice site usage, and splice junction strength, at single-base-pair resolution [195]. Trained on human and mouse genomes, the model matches or exceeds specialist models in 25 of 26 variant effect benchmarks. For neurological disease, its simultaneous multi-modal scoring of regulatory variants enables the identification of a single non-coding variant that disrupts a CCCTC-binding factor (CTCF) binding site, alters neuronal chromatin accessibility, and reduces expression of a disease-relevant gene; this represents a qualitative advance over single-modality interpretation.

Together, these models form a progression from accurate sequence representation, through long-range regulatory context, to population-scale variant interpretation, to integrated multi-modal functional prediction. Collectively, they provide tools to decode the non-coding regulatory landscape that governs brain development and disease.

## Protein FMs

Proteins are the primary effectors of neurological function and disease: the misfolding of tau, amyloid-beta, and alpha-synuclein drives neurodegeneration, while the structural integrity of synaptic receptors, ion channels, and brain-resident immune proteins determines circuit function and inflammatory state. Protein FMs have transformed the field by learning the evolutionary and structural grammar of protein sequences from hundreds of millions of examples, enabling structure prediction, variant effect analysis, and peptide property prediction at scales previously inaccessible to experimental methods.

AlphaFold2 and AlphaFold3, developed by Google DeepMind, represent successive breakthroughs in structural biology with direct relevance to neurological disease. AlphaFold2 achieved atomic-accuracy prediction of protein 3D structures from sequence alone [196], winning CASP14 and earning the 2024 Nobel Prize in Chemistry; its open-source structural database has enabled mechanistic studies of neurodegeneration, including prediction of the Aβ42 monomer-to-hexamer aggregation pathway in AD [218]. AlphaFold3 extended this framework to unified prediction of biomolecular complexes through an integrated diffusion-based architecture [197], and has been applied to characterize structural consequences of 7 AD-associated missense pQTL variants across microglial proteins, including TREM2, CD33, and PILRB. Where AlphaFold models predict static structures, ESM-2 and ESMFold learn the evolutionary language of protein sequences directly from 250 million sequences spanning 86 billion amino acids, enabling structure prediction and variant effect scoring at 60× the speed of AlphaFold2 [219]. For neurodegeneration, ESM-2 embeddings have been applied to predict aggregation propensity and phase separation behavior of tau, APP, and alpha-synuclein [198]. Complementing this, AlphaMissense classified 71 million possible human missense variants by combining AlphaFold2 structural representations with evolutionary constraint signals [199], providing systematic pathogenicity scores across neurodegeneration-associated genes, including LRRK2, SNCA, APP, and PSEN1. A distinct class of protein FMs addresses quantitative brain proteomics. Prosit predicts peptide fragment ion intensities and retention times, enabling DIA workflows with more than tenfold reductions in false discovery rate and substantially deeper proteome coverage in brain tissue studies [220]. AlphaPeptDeep extends this to simultaneous prediction of retention time, collisional cross section, and MS2 spectra, with transfer learning enabling rapid adaptation to novel PTMs critical for studying synaptic protein phosphorylation and ubiquitination in disease [200]. DeepLC complements both by providing zero-shot retention time prediction for PTMs absent from its training data, enabling unbiased discovery of novel modification sites on neurological disease proteins [221].

Together, these models form a coherent pipeline from atomic structure prediction and variant pathogenicity classification to deep quantitative proteome profiling—collectively decoding neurological disease mechanisms at the protein level.

## Single-cell FMs

Understanding the cellular diversity of the human brain requires more than cataloguing gene expression: it demands representations that can annotate hundreds of cell types, predict responses to perturbations, and generalize across datasets, species, and disease states. Single-cell foundation models (scFMs) address this challenge by treating genes as tokens and cells as sentences, learning context-dependent gene interaction patterns from millions of transcriptomes through self-supervised pretraining.

scGPT pioneered the GPT-style generative architecture for single-cell data, pretraining on over 33 million cells across 441 studies and 51 human organs [201]. Fine-tuned on HBCA data for perirhinal cortex annotation, scGPT demonstrated that general-purpose pretraining could produce representations directly applicable to the specialized cellular taxonomy of the brain. Its batch integration capabilities provide a practical tool for harmonizing the multi-site transcriptomic datasets that characterize large-scale brain atlas efforts. While scGPT established the generative paradigm, Geneformer extended its interpretive power through in silico perturbation. Employing BERT-style masked language modeling, Geneformer’s key contribution to neuroscience lies in its capacity to predict the transcriptional consequences of silencing or overexpressing specific genes in silico, without the need for corresponding experimental data [202]. This capability has been applied to dosage sensitivity analysis of disease-associated genes in neurons and fetal cerebrum, contexts where generating matched perturbation data is both technically demanding and ethically constrained.

Complementing Geneformer’s perturbation modeling, scFoundation brought architectural innovation to the challenge of expression resolution. Its asymmetric transformer with 100 million parameters and direct value projection preserves full expression magnitudes rather than binarized rank data, enabling prediction of cell-type-specific drug responses with high area under the precision-recall curve (AUPR) [203]. For brain pharmacology, this offers a computational route to model CNS drug effects across the heterogeneous cellular landscape of the human brain—a critical capability given the difficulty of obtaining matched human brain tissue for drug testing. Building on this cross-species capability, GeneCompass introduces knowledge-informed pretraining that integrates gene regulatory networks, promoter sequences, and transcription factor–target relationships alongside expression data. Trained on 120 million cells from human and mouse, GeneCompass demonstrates superior cross-species cell type annotation on brain datasets [205]. Its incorporation of regulatory logic rather than expression alone offers a natural fit for neuroscience, where transcription factor programs define and maintain cell identity throughout life and disease. CellFM, currently the largest scFM with 800 million parameters trained on 100 million human cells using a modified RetNet framework, exemplifies how scale directly translates to brain research utility [206]. Its superior performance in cell annotation, perturbation prediction, and gene function prediction specifically benefits rare brain cell populations, such as interneuron subtypes, specialized astrocytes, and disease-associated microglia. While these populations are often underrepresented in smaller training corpora, they frequently represent the most biologically and clinically informative elements in the study of neurological disease.

Across these models, a clear developmental arc emerges: from pioneering generative architecture through mechanistic perturbation modeling, pharmacological prediction, cross-species generalization, regulatory knowledge integration, and finally the scale required to represent the full complexity of brain cellular diversity. For large-scale brain cell-type annotation, scGPT and CellFM are recommended for practical model selection. Specifically, the 100-million-cell human brain training corpus of CellFM provides substantial advantages for identifying rare interneuron and disease-associated microglial subtypes. Alternatively, Geneformer is preferred for in silico perturbation modeling in neurons when matched experimental data are unavailable. It is critical for researchers to consider that scFoundation and UCE were pre-trained predominantly on non-brain or bulk tissue data. Consequently, their performance on brain-specific rare cell populations requires independent validation prior to deployment in atlas construction or clinical applications.

## Spatial omics FMs

Dissociation-based single-cell methods sacrifice a fundamental dimension of brain organization: tissue architecture. The laminar structure of the cortex, the trisynaptic circuit of the hippocampus, and the perivascular niches of brain-resident immune cells: these spatial relationships are not merely anatomical decorations but are mechanistically constitutive of brain function. Spatial omics FMs restore this context, learning representations that integrate transcriptional identity with tissue topology.

Nicheformer was the first FM to train jointly on dissociated single-cell and spatially resolved transcriptomics data, pretraining on SpatialCorpus-110M, over 57 million dissociated and 53 million spatially resolved cells across 73 tissues [208]. By demonstrating that models trained solely on dissociated data systematically fail to recover the complexity of tissue microenvironments, Nicheformer established a key principle for brain atlas construction: spatial context is not optional. Its zero-shot transfer across spatial platforms makes it immediately applicable to the heterogeneous landscape of brain spatial transcriptomic datasets. Extending spatial generalization further, Novae introduced a graph neural network-based architecture trained on approximately 30 million spatial transcriptomics cells, with the distinctive capability of zero-shot domain inference across unseen gene panels and technologies [209]. Validated against the Allen Reference Atlas on mouse brain sections, Novae accurately recovers hierarchical neuroanatomical boundaries, a technology-agnostic parcellation capability essential for integrating brain datasets generated on different spatial platforms, which currently remain difficult to compare directly. Where Novae parcellates based on transcriptional domains, OmiCLIP bridges modalities: by pioneering contrastive learning between H&E histology images and spatial transcriptomic profiles across 2.2 million paired samples from 32 organs, OmiCLIP enables prediction of molecular spatial organization directly from tissue morphology [210]. For neuroscience, this capability is transformative: it makes molecular-resolution spatial analysis accessible to the vast archives of neuropathology tissue, including historical samples from AD, PD, and other neurological diseases that predate spatial sequencing technologies by decades.

scGPT-spatial and OmniCell represent the current frontier of spatial modeling, tackling the practical demands of multi-platform brain atlas construction from complementary directions. scGPT-spatial extends the scGPT framework through continual pretraining on SpatialHuman30M (30 million profiles from Visium, Visium HD, MERFISH, and Xenium platforms across 821 tissue slides), with a mixture-of-experts decoder that enables protocol-aware gene expression decoding [211]. OmniCell takes a structurally distinct approach: as the first model to jointly represent intra-cellular gene expression and inter-cellular spatial dependencies within a unified architecture, it serializes spatially adjacent cells as context during training, learning representations that simultaneously encode transcriptional state and tissue topology [212]. Benchmarked on a MERFISH mouse brain aging atlas spanning 31 datasets, OmniCell outperforms scGPT-spatial and Nicheformer on spatial clustering and achieves rare cell detection accuracy 13 percentage points above competing models, a margin with direct implications for detecting the sparse, spatially restricted cell populations most relevant to neurological disease. For spatial brain applications, OmniCell and scGPT-spatial are recommended for tasks requiring the preservation of tissue architecture and spatial dependencies. However, their performance has been most extensively validated on mouse brain datasets, suggesting that human applications require further benchmarking. Regarding regional analysis, Nicheformer and Novae are preferred for cross-platform niche characterization and brain region parcellation, respectively. Researchers should exercise caution as both models remain sensitive to variations in gene panels and platform-specific characteristics.

## Medical LLMs: accelerating neurological disease diagnosis

The translation of molecular insights into clinical benefit requires bridging the gap between biological complexity and medical reasoning. Medical large language models have achieved this at scale, reaching clinician-level performance on standardized medical examination benchmarks and enabling new approaches to diagnosis, drug repurposing, and clinical text analysis in neurological disease.

Med-PaLM2 from Google was the first AI system to achieve expert-level performance on MedQA [213], with 86.5% accuracy and responses preferred over physician answers on 8 of 9 clinical evaluation axes. Its multimodal successor, Med-PaLM M, integrates imaging, clinical text, and genomic data in a unified framework, a capability directly applicable to the multimodal nature of neurological case analysis, where diagnosis typically depends on the synthesis of MRI findings, clinical presentation, biomarker profiles, and genetic information. GatorTron, developed by the University of Florida and NVIDIA, scales clinical language modeling to its current frontier: 8.9 billion parameters trained on over 90 billion words, including 82 billion words of de-identified clinical notes [214]. Its generative successor GatorTronGPT extends to 20 billion parameters and demonstrates 9.6% improvement on natural language inference tasks. For neurology, GatorTron’s deep grounding in clinical documentation enables the extraction of structured neurological information from the unstructured free text that constitutes the majority of clinical knowledge. This structured data includes critical clinical elements such as symptom timelines, medication responses, and markers of disease progression. Complementing these institutional systems, Meditron from EPFL and Yale provides the leading open-source alternative: a Llama-2-based model (7B and 70B versions) achieving 77.6% accuracy on MedQA, within 1% of GPT-4, trained on the GAP-Replay corpus of 48.1 billion tokens, including 46,000 clinical practice guidelines. Meditron’s open availability makes it a critical resource for academic neurology research, where access to proprietary clinical LLMs is constrained.

The clinical impact of these models is already materializing in neurological disease. LLM-driven drug repurposing analyses, validated on real-world data from the Vanderbilt and All of Us cohorts, have identified metformin, simvastatin, and losartan as associated with reduced AD risk [222]. Multi-source LLM integration achieves 0.849 F1 for glioblastoma presence classification and 0.929 for tumor stability assessment from radiology reports. At the regulatory frontier, the FDA has cleared AI-powered neurology devices, including icobrain aria, which represents the first AI software designed to detect and grade amyloid-related imaging abnormalities (ARIAs). This capability is critical for monitoring patients receiving anti-amyloid therapies such as lecanemab. Alongside this, NeuroQuant 5.0 provides deep learning-based brain segmentation integrated with ARIA detection to enhance AD monitoring and clinical decision-making. These approvals signal a shift from research demonstration to clinical deployment, marking the beginning of AI-assisted neurological care at scale.

## Convergence toward multimodal integration

The most significant trend is convergence toward multimodal integration. Models like Genos pair 10B parameter genomic encoders with 4B parameter language models for omics-text reasoning. UCE leverages ESM-2 protein embeddings to enable cross-species cell type annotation. Nicheformer jointly trains on dissociated and spatial data with organism and assay tokens enabling transfer across modalities. CAPTAIN [207] exemplifies a new generation of multimodal scFMs pretrained on over 4 million cells with concurrently measured transcriptomes and a curated repertoire of 382 surface proteins, learning unified representations by explicitly modeling cross-modality dependencies between RNA and protein; the model uncovers protein-driven intercellular dynamics, including immune interaction patterns linked to COVID-19 severity. Similarly, OmniCell unifies scRNA-seq and spatial transcriptomics by serializing spatial neighborhood graphs within a shared Transformer architecture pretrained on 67 million cells, enabling seamless transfer across modalities and platforms. At the genomic level, AlphaGenome integrates predictions across 8 functional modalities (gene expression, splicing, chromatin accessibility, histone modifications, transcription factor binding, chromatin contacts, and splice junction coordinates) from a single unified model, enabling multi-dimensional scoring of regulatory variants relevant to neurological disease. For brain multi-omics specifically, these capabilities translate to substantially expand the analytical scope. gFMs predict variant effects in neurological disease genes with >90% accuracy. Single-cell models trained on 100+ million cells enable automated brain cell type annotation and perturbation prediction. Spatial models preserve the 3-dimensional organization critical to understanding neural circuits. Medical LLMs synthesize clinical literature and patient records for diagnosis and treatment optimization.

The path to clinical translation is accelerating. Models like Genos and Meditron are released under open-source licenses enabling academic validation. FDA clearances for brain-specific AI tools have reached approximately 30 devices. The integration of FM predictions with established brain atlases from Allen Brain Institute, Human Cell Atlas, and the BRAIN Initiative Cell Atlas Network (BICAN) provides the validation infrastructure necessary for clinical deployment. As these models continue scaling, with the largest now exceeding 40 billion parameters and training datasets approaching 10 trillion bases, their emergent capabilities will increasingly define the frontier of computational neuroscience and precision neurology.

### Clinical applications of AI-driven multi-omics integration in neurological diseases

The sophisticated AI methodologies described above provide the essential computational infrastructure for addressing one of the most pressing challenges in precision neurology: the accurate stratification of clinically heterogeneous brain disorders into biologically meaningful subtypes. Traditional diagnostic frameworks, predominantly reliant on clinical symptomatology and gross imaging features, frequently fail to capture the underlying molecular heterogeneity that drives variable disease trajectories and differential treatment responses. The integration of AI-driven multi-omics analysis fundamentally transforms this paradigm by enabling data-driven disease reclassification based on comprehensive molecular signatures rather than phenomenological observations alone.

Specifically, machine learning classifiers based on single-cell atlases can identify disease-specific cell subpopulations and molecular biomarkers from patient peripheral blood samples [223]. Research focused on AD has developed classification models based on single-cell transcriptomic data, whereby analysis of gene expression patterns in peripheral blood mononuclear cells enables differentiation between AD patients and healthy controls with considerable accuracy [223, 224]. Regarding prognostic prediction, AI models can extract prognosis-related features from patient multi-omics data to forecast disease progression rates and treatment responses. Research on glioblastoma has utilized single-cell transcriptomic data to identify tumor cell subpopulations associated with poor prognosis [225, 226], with these cells exhibiting stem cell-like characteristics and elevated expression of therapy resistance-associated genes. Predictive models based on the abundance of these cell subpopulations can accurately forecast patient survival, providing evidence for individualized treatment decisions. Time-series deep learning models, including recurrent neural networks and Transformer architectures, have been applied to analyze longitudinal multi-omics data, capturing the dynamic evolution of disease and predicting disease trajectories.

In drug target discovery, AI-driven multi-omics analysis enables systematic identification of key disease drivers and potential therapeutic targets [227, 228]. Gene regulatory network inference methodologies [229] identify aberrantly activated transcription factors and signaling pathways in disease states, with these molecules representing potential therapeutic targets. Cell–cell communication analysis has revealed abnormal ligand-receptor interactions within the disease microenvironment [230], providing insights for therapeutic strategies targeting intercellular communication. Deep learning-based drug repositioning approaches integrate multi-omics data with drug-target interaction databases to predict the potential efficacy of existing drugs against brain disorders, thereby accelerating drug development processes. Implementation of precision medicine requires understanding inter-patient heterogeneity and formulating individualized treatment regimens. Single-cell multi-omics profiling enables molecular stratification of patients into distinct disease subtypes with differential treatment responses [231, 232]. For instance, in glioblastoma, integration of single-cell transcriptomics and chromatin accessibility data has identified patient subgroups characterized by distinct cellular compositions and regulatory landscapes, which correlate with differential responses to immunotherapy and targeted therapies [231, 233]. Machine learning models trained on patient-specific multi-omics signatures can predict individual responses to therapeutic interventions, enabling clinicians to prioritize treatment options with the highest likelihood of efficacy while minimizing adverse effects. Furthermore, longitudinal monitoring of circulating biomarkers through liquid biopsy approaches, coupled with AI-driven trajectory analysis, facilitates real-time assessment of treatment efficacy and early detection of therapeutic resistance, thereby enabling dynamic treatment adjustments in clinical practice [231, 232].

### From laboratory to clinical: challenges and perspectives

Despite the considerable potential demonstrated by multi-omics AI technologies in brain research, the translation from laboratory investigation to clinical application continues to face multiple challenges.

The foremost challenge concerns data quality and standardization. Single-cell and spatial transcriptomic data exhibit substantial technical batch effects [234, 235], rendering data generated across different platforms and laboratories difficult to compare directly [236]. Establishing standardized protocols for data acquisition, processing, and quality control constitutes the foundation for enabling data sharing and cross-study comparisons. International collaborative initiatives such as the Human Cell Atlas [237] and the Brain Initiative are actively promoting data standardization and open sharing practices, thereby establishing a foundation for field advancement. This includes adoption of unified metadata schemas (e.g., BICAN metadata standards), consensus cell type nomenclature systems, and platform-agnostic quality control benchmarks. The lack of standardized ontologies for brain cell types across studies remains a particular barrier, as different atlases use inconsistent cell type labels and hierarchical classifications, hampering meta-analysis and cross-study replication. Besides, clinical validation and regulatory approval represent the final critical steps in technology translation. AI diagnostic and prognostic tools require validation of their efficacy and safety through large-scale, multicenter clinical studies [238], demanding substantial investments of time and resources. Regulatory agencies such as the Food and Drug Administration are establishing approval frameworks for AI medical devices, although specific standards for multi-omics AI tools remain under development. Strengthening collaborations across academia, industry, healthcare, and research institutions, and establishing clinical validation platforms and translational research centers, represent effective strategies for accelerating clinical translation of technologies. As well, the interpretability and trustworthiness of AI models constitute critical requirements for clinical implementation. Explainable AI methodologies, including attention visualization, feature importance analysis, and causal inference techniques, are under active development, aiming to elucidate the biological foundations underlying model decisions. The development of AI architectures with inherent interpretability, such as graph neural networks informed by biological prior knowledge and symbolic reasoning systems, represents an important direction for enhancing model trustworthiness [239]. Notwithstanding the above challenges, the field has achieved important breakthroughs. Multiple AI-based medical imaging diagnostic systems have received regulatory approval and been implemented in clinical practice, establishing precedents for brain disease AI diagnostics. The cost of single-cell sequencing continues to decline, and commercial platforms are becoming increasingly mature [240], enabling multi-omics technologies to gradually transition from research tools to clinical diagnostic applications. Several early diagnostic products for brain disease based on liquid biopsy and multi-omics biomarkers are currently in clinical trial phases, demonstrating promising diagnostic performance. With continued technological advancement, establishment of standards, and refinement of regulatory frameworks, within the next 5–10 years, provided that prospective multicenter clinical validation studies are completed, regulatory-grade evidence standards are met, and reimbursement pathways are established. These conditions represent non-trivial barriers. For context, AI diagnostic tools for radiology, which is a technically simpler domain, have required over a decade to move from proof of concept to routine clinical deployment.

### Looking ahead: the next decade of brain disease research

#### Technological frontiers and future directions

Over the next decade, brain science research is positioned to achieve breakthrough advances across technological, theoretical, and applied dimensions. At the technological level, single-cell multi-omics methodologies will advance toward higher throughput, reduced costs, and more comprehensive molecular coverage. Spatial multi-omics technologies are expected to achieve genuine single-cell and even subcellular resolution, enabling simultaneous detection of tens of thousands of genes, proteins, and metabolites. Advances in live imaging technologies will enable researchers to observe brain activity and molecular dynamics in real time without tissue damage, while integration with optogenetic and chemogenetic approaches will facilitate precise neural circuit manipulation.

A particularly important emerging resource is the neurological digital twin, a patient-specific computational model that integrates multimodal data to simulate disease mechanisms, predict trajectories, and test interventions in silico. In epilepsy, the Virtual Brain and Virtual Epileptic Patient frameworks use individual structural MRI, diffusion MRI, and SEEG/EEG recordings to model seizure initiation and propagation [241, 242]. Subsequent studies further showed that these models can estimate epileptogenic zones, relate simulated networks to surgical outcomes, and support presurgical evaluation in drug-resistant epilepsy [243, 244]. In AD, digital-twin and disease-progression models have been used to simulate biomarker and cognitive trajectories, support drug-discovery strategies, and improve clinical-trial efficiency by estimating individualized placebo trajectories [245–247]. These examples indicate that digital twins may link brain atlases, longitudinal biomarkers, and individualized intervention planning, although their integration with single-cell, spatial, and multi-omics datasets still requires standardized data structures, interpretable models, and prospective clinical validation.

In the domain of AI methodologies, the concepts of FMs and large language models are expanding into the biomedical field with remarkable momentum. Recent years have witnessed the emergence of specialized FMs for biological sequence analysis, including scGPT [201], Geneformer [202], etc. These general-purpose brain science models pretrained on massive multi-omics datasets can be applied to diverse downstream tasks through transfer learning, including cell type identification, disease diagnosis, and drug response prediction, substantially reducing the data requirements and training costs associated with specific applications. This convergence of large-scale pretraining, transfer learning, and hybrid reasoning architectures positions AI-driven multi-omics analysis at the forefront of precision neurology advancement.

#### Data standardization and harmonization

Data standardization is a prerequisite for translating brain multi-omics atlases from descriptive resources into reusable analytical and clinical frameworks. Brain datasets are often generated across different donors, anatomical regions, disease states, laboratories, sequencing chemistries, and computational pipelines, making them vulnerable to batch effects that may be misinterpreted as biological variation. Several integration methods, including mutual-nearest-neighbor correction, Harmony, and anchor-based single-cell integration, have improved cross-dataset comparability, but these methods cannot substitute for standardized sample processing, quality control, and metadata reporting [117, 234, 248]. Spatial transcriptomic datasets require additional spatially informed integration strategies. For example, spatiAlign uses unsupervised contrastive learning to integrate gene-expression profiles with spatial coordinates, enabling batch-effect correction and joint analysis of multiple spatial transcriptomic sections, including time-series brain sections [249].

Large international initiatives provide useful models for harmonization. The Human Cell Atlas emphasizes common experimental, computational, and data-sharing principles for constructing interoperable cellular reference maps, while the BICCN has demonstrated the value of coordinated multimodal profiling for defining reproducible brain cell taxonomies across transcriptomic, epigenomic, anatomical, and physiological modalities [99, 117]. Future brain multi-omics studies should therefore report core metadata, including donor characteristics, anatomical region, disease status, postmortem interval or sampling procedure, tissue preservation, nuclei or cell isolation protocols, sequencing platform, read depth, quality-control thresholds, normalization strategy, integration method, and batch covariates. Adoption of FAIR data principles would further improve data findability, interoperability, and reusability, enabling independent validation and cross-cohort comparison [250].

#### Ethical considerations in large-scale brain multi-omics research

Large-scale brain multi-omics research raises ethical concerns because genomic, epigenomic, transcriptomic, spatial, and clinical metadata can carry re-identification risks even when conventional identifiers are removed. Previous studies have shown that genomic information can enable identity inference through surname prediction or familial matching, highlighting the limits of simple de-identification strategies [251–253]. Therefore, informed consent should clearly describe future data use, data-sharing scope, controlled-access mechanisms, potential privacy risks, withdrawal options, and governance procedures. Responsible data sharing should combine participant-centered consent, transparent access review, data-use agreements, audit trails, and privacy-preserving computational approaches where appropriate [254, 255].

Equity is another central issue in global brain atlas construction. If reference atlases are disproportionately derived from specific ancestries, regions, or health-care systems, downstream biological interpretation and clinical tools may generalize poorly to underrepresented populations. This concern is well recognized in human genomics, where a lack of diversity can limit discovery and worsen inequities in precision medicine [256, 257]. Ethical atlas construction should therefore include diverse populations, community engagement, equitable authorship and data-access policies, and capacity building across regions. Finally, AI-driven diagnostic tools trained on multi-omics data require careful oversight. Clinical algorithms can reproduce or amplify existing inequities when training data, labels, or deployment settings are biased, so models should be externally validated across ancestry, sex, age, disease subtype, and acquisition site, with transparent reporting, clinician oversight, and post-deployment monitoring [258–260].

#### The establishment of the brain project international grand science alliance

Given the complexity and global scope of brain science, international collaborations across major brain initiatives have progressively strengthened. National-level projects such as the US BICCN [91, 261], the European Union’s Human Brain Project [262], China Brain Project [263], and Japan’s Brain/MINDS [264] have been underway for many years, yielding important results. In September 2025, the International Consortium for Primate Brain Mapping (ICPBM) was officially established in Shanghai by the Chinese Academy of Sciences, the BGI research group, and the University of Hainan, marking a new phase of global collaboration in brain research. As one of the organizers, BGI research group provides the up-to-date multi-single cell sequencing and spatial transcriptomic technologies, as well as AI computing power and resources. Currently, ICPBM brings together over one hundred scientists from 25 countries with a 25-year roadmap to systematically map the multi-omics atlases of marmoset, macaque, and human brains. This comprehensive mapping aims to integrate cell-type classifications with gene expression patterns and projection connectivity. *Science* magazine highlighted the initiative, noting that its scale will have an enormous impact on the field. The consortium chair, Professor Mu-ming Poo, emphasized that by promoting data sharing and standard unification, this initiative will provide crucial insights into the molecular mechanisms of neurological diseases ranging from stroke to AD.

Significantly, ICPBM complements rather than duplicates existing initiatives. While BICCN has primarily focused on mouse and human brain cell census with cross-species comparisons, and the Human Brain Project has emphasized large-scale computational simulation and European cohort integration, ICPBM’s distinctive contribution lies in its systematic multi-omics mapping of non-human primate brains. By focusing on marmosets and macaques over a 25-year horizon, the consortium fills a critical gap in the translational bridge between rodent models and human pathophysiology. This long-term primate focus is particularly valuable for neurological research, where differences between humans and mice in disease-relevant cell types have traditionally acted as significant translational barriers. Looking forward, the synergy of technological advances, AI development, and deepening global cooperation suggests that the next decade will witness revolutionary breakthroughs. Just as the Human Genome Project ushered in the era of genomic medicine, brain cell atlas projects like ICPBM are now opening a new era of precision neurology.

## Additional files

**Supplementary Table S1:** Overview of Brain Atlas Projects (Species).

## List of abbreviations

ABC: Allen Brain Cell Atlas; AD: Alzheimer’s disease; ADHD: attention deficit hyperactivity disorder; AI: artificial intelligence; ALS: amyotrophic lateral sclerosis; AMP-AD: Accelerating Medicines Partnership-Alzheimer’s Disease; ASAP-PMDBS: Aligning Science Across Parkinson’s–Parkinson’s Disease Molecular Brain Study; ASD: autism spectrum disorder; ATAC: Assay for Transposase-Accessible Chromatin; ATAC-seq: Assay for Transposase-Accessible Chromatin using sequencing; BICAN: BRAIN Initiative Cell Atlas Network; BICCN: BRAIN Initiative Cell Census Network; CEL-seq: Cell Expression by Linear amplification and Sequencing; CH-ATAC-seq: combinatorial-hybridization-based ATAC-seq; Chip-Tip: single-cell proteomics workflow; CITE-seq: Cellular Indexing of Transcriptomes and Epitopes by sequencing; DBiT-seq: Deterministic Barcoding in Tissue for spatial omics sequencing; DNB: DNA nanoball; DNBelab C4: DNA nanoball-based single-cell sequencing platform; Drop-seq: droplet-based single-cell RNA sequencing; FDA: Food and Drug Administration; HCA: Human Cell Atlas; HBCA: Human Brain Cell Atlas; ICPBM: International Consortium for Primate Brain Mapping; MALBAC: multiple annealing and looping-based amplification cycles; MDA: multiple displacement amplification; MERFISH: multiplexed error-robust fluorescence *in situ* hybridization; MIBI: multiplexed ion beam imaging; MINDS: Marmoset Brain Mapping by Integrated Neurotechnologies for Disease Studies; MRI: magnetic resonance imaging; MS: multiple sclerosis; PD: Parkinson’s disease; PsychENCODE: Psychiatric Encyclopedia of DNA Elements; scATAC-seq: single-cell ATAC-seq; scCAT-seq: single-cell chromatin accessibility and transcriptome sequencing; scMEP: single-cell metabolic profiling; scRNA-seq: single-cell RNA sequencing; scSpaMet: single-cell spatial metabolomics; sci-ATAC-seq: single-cell combinatorial indexing ATAC-seq; SCZ: schizophrenia; SEAM: spatial single nuclear metabolomics method; SEA-AD: Seattle Alzheimer’s Disease Brain Cell Atlas; SHARE-seq: Simultaneous High-throughput ATAC and RNA Expression with sequencing; SIDR: simultaneous isolation and parallel sequencing of genomic DNA and total RNA; Slide-seq: bead-based spatial transcriptomic sequencing; SMART-seq2: Switching Mechanism at 5' End of RNA Template sequencing 2; SM-Omics: spatial multi-omics; snATAC-seq: single-nucleus ATAC-seq; snMultiome: single-nucleus multiome sequencing; snRNA-seq: single-nucleus RNA sequencing; Spatial-ATAC-seq: spatial ATAC-seq; Spatial ATAC-RNA-seq: spatial joint profiling of chromatin accessibility and RNA; Spatial-CUT&Tag: spatial cleavage under targets and tagmentation; Spatial CITE-seq: spatial CITE-seq; SPLIT-seq: split-pool ligation-based transcriptome sequencing; STARmap: spatially resolved transcript amplicon readout mapping; TBI: traumatic brain injury.

## Supplementary Material

giag075_supplementary_table1

giag075_Authors_Response_To_Reviewer_Comments_Original_Submission

giag075_GIGA-D-26-00090_Original_Submission

giag075_GIGA-D-26-00090_Revision_1

giag075_Reviewer_1_Report_Original_Submission Reviewer 1 -- 4/13/2026

giag075_Reviewer_1_Report_Revision_1 Reviewer 1 -- 6/11/2026

giag075_Reviewer_2_Report_Original_Submission Reviewer 2 -- 4/20/2026

giag075_Reviewer_2_Report_Revision_1 Reviewer 2 -- 6/11/2026

## Data Availability

No data are associated with this article.
