# Supplementary material for: From the brain cell atlas to precision neurology: a review of the application of AI-driven multi-omics in brain science
Source: Gigascience. 2026 Jun 23;15:giag075. doi: 10.1093/gigascience/giag075 (PMC13354932; doi:10.1093/gigascience/giag075)
Supplement: giag075_GIGA-D-26-00090_Original_Submission [file giag075_giga-d-26-00090_original_submission.pdf]

GigaScience

From the Brain Cell Atlas to Precision Neurology: A review of the application of AI-driven multi-omics in brain science

--Manuscript Draft--

**Manuscript Number:**	GIGA-D-26-00090
**Full Title:**	From the Brain Cell Atlas to Precision Neurology: A review of the application of AI-driven multi-omics in brain science
**Article Type:**	Review
**Funding Information:**	National Key Research and Development (R&D) Program (2021YFA0805100) — professor Shiping Liu<br>Key Program of the National Natural Science Foundation of China (32530027) — professor Lifang Wang<br>National Science and Technology Innovation 2030 Major Program (2021ZD0204400) — professor Lei Han
**Abstract:**	Recent advances in multi-omics technologies have catalyzed the construction of comprehensive brain cell atlases, providing essential data foundations for artificial intelligence (AI)-driven analyses in precision neurology. This review systematically examines how the integration of AI with single-cell multi-omics and spatial multi-omics enables unprecedented resolution in deciphering brain cellular architecture across health and disease states. Through systematic evaluation of multi-omics datasets from neurodegenerative, psychiatric, and neurodevelopmental disorders, we demonstrate how AI facilitates disease subtype stratification, biomarker discovery, and therapeutic target identification. We critically address translational challenges, including data standardization, model interpretability, and regulatory frameworks for clinical implementation. Notably, the establishment of the International Consortium for Primate Brain Mapping (ICPBM) in 2024 exemplifies ongoing global collaborative efforts toward systematic multi-omics atlas construction across species and disease states. This synthesis underscores a paradigm shift toward AI-enabled, mechanism-driven analyses, ultimately positioning precision neurology as a realizable framework for individualized diagnosis and targeted interventions in complex brain disorders.
**Corresponding Author:**	Lifang Wang<br>BGI Research, Hangzhou<br>Hangzhou, CHINA
**Corresponding Author Secondary Information:**	
**Corresponding Author's Institution:**	BGI Research, Hangzhou
**Corresponding Author's Secondary Institution:**	
**First Author:**	Youzhe He
**First Author Secondary Information:**	
**Order of Authors:**	Youzhe He<br>Yanrong Wei<br>Jingxi Zhi<br>Chunyu Huang<br>Lei Han<br>Shiping Liu<br>Lifang Wang
**Order of Authors Secondary Information:**	

Powered by Editorial Manager® and ProduXion Manager® from Aries Systems Corporation

Additional Information:	
**Question**	**Response**
Are you submitting this manuscript to a special series or article collection?	No
**Experimental design and statistics**<br><br>Full details of the experimental design and statistical methods used should be given in the Methods section, as detailed in our Minimum Standards Reporting Checklist. Information essential to interpreting the data presented should be made available in the figure legends.<br><br>Have you included all the information requested in your manuscript?	Yes
**Resources**<br><br>A description of all resources used, including antibodies, cell lines, animals and software tools, with enough information to allow them to be uniquely identified, should be included in the Methods section. Authors are strongly encouraged to cite Research Resource Identifiers (RRIDs) for antibodies, model organisms and tools, where possible.<br><br>Have you included the information requested as detailed in our Minimum Standards Reporting Checklist?	No
If not, please give reasons for any omissions below.<br><br>as follow-up to "**Resources**<br><br>A description of all resources used, including antibodies, cell lines, animals and software tools, with enough information to allow them to be uniquely identified, should be included in the Methods section. Authors are strongly	This manuscript is a review article that synthesizes and discusses previously published studies. It does not involve the generation of new experimental data, and therefore does not include original use of antibodies, cell lines, animals, or novel software tools.

Powered by Editorial Manager® and ProduXion Manager® from Aries Systems Corporation

encouraged to cite Research Resource Identifiers (RRIDs) for antibodies, model organisms and tools, where possible.<br><br>Have you included the information requested as detailed in our Minimum Standards Reporting Checklist?<br><br>"	
**Availability of data and materials**<br><br>All datasets and code on which the conclusions of the paper rely must be either included in your submission or deposited in publicly available repositories (where available and ethically appropriate), referencing such data using a unique identifier in the references and in the "Availability of Data and Materials" section of your manuscript.<br><br>Have you have met the above requirement as detailed in our Minimum Standards Reporting Checklist?	No
If not, please give reasons for any omissions below.<br><br>as follow-up to "**Availability of data and materials**<br><br>All datasets and code on which the conclusions of the paper rely must be either included in your submission or deposited in publicly available repositories (where available and ethically appropriate), referencing such data using a unique identifier in the references and in the "Availability of Data and Materials" section of your manuscript.<br><br>Have you have met the above requirement as detailed in our Minimum Standards Reporting Checklist?	This manuscript is a review article and does not generate or analyze any new datasets or original code. All data and findings discussed are derived from previously published literature, which is fully cited within the reference list. Therefore, there are no new datasets or code to deposit in a publicly available repository.

"	
GigaScience has policies and guidelines in place for the use of generative AI-writing tools such as ChatGPT. If you have used such writing tools to assist with writing the manuscript this must be declared and cited in the text. Authors should not list AI-writing tools and other AI-assisted technologies as an author or co-author and should acknowledge that they are fully responsible for text generated or refined by AI-writing tools.<p><br><br>A summary of use (particularly in the introduction or among methods) needs to be included at the end of the paper, and the outputs should also be included as a supplementary file hosted in GigaDB or other open repositories. Please read our guidelines for more information. <p><br><br>By submitting to GigaScience, you are aware of the journal's AI-writing tools policy, and if you have declared use of such tools below, you have acknowledged this where appropriate in your manuscript and have made a summary of use and outputs available. <p><br><b>AI-assisted writing tools have been used in the preparation of this manuscript?	Yes

Powered by Editorial Manager® and ProduXion Manager® from Aries Systems Corporation

Manuscript
Click here to access/download;Manuscript;Review-Gigascience-260310.docx

From the Brain Cell Atlas to Precision Neurology: A review of the application of AI-driven multi-omics in brain science

Youzhe He[1,2†], Yanrong Wei[1,2†], Jingxi Zhi[2,3], Chunyu Huang[1,2], Lei Han[4,5], Shiping Liu[1,5], Lifang Wang[2,4*]

Author affiliations:

[1] College of Life Sciences, University of Chinese Academy of Sciences, Beijing 100049, China.
[2] BGI Research, Hangzhou 310030, China.
[3] School of Life Science, Hangzhou Institute for Advanced Study, University of Chinese Academy of Sciences, Hangzhou 310030, China
[4] BGI Research, Shenzhen 518083, China.
[5] State Key Laboratory of Genome and Multi-omics Technologies, BGI Research, Hangzhou 310030, China.

[†]These authors contributed equally to this work.
*Corresponding author. Email: Wanglifang@genomics.cn

Abstract

Recent advances in multi-omics technologies have catalyzed the construction of comprehensive brain cell atlases, providing essential data foundations for artificial intelligence (AI)-driven analyses in precision neurology. This review systematically examines how the integration of AI with single-cell multi-omics and spatial multi-omics enables unprecedented resolution in deciphering brain cellular architecture across health and disease states. Through systematic evaluation of multi-omics datasets from neurodegenerative, psychiatric, and neurodevelopmental disorders, we demonstrate how AI facilitates disease subtype stratification, biomarker discovery, and therapeutic target identification. We critically address translational challenges, including data standardization, model interpretability, and regulatory frameworks for clinical implementation. Notably, the establishment of the International Consortium for Primate Brain Mapping (ICPBM) in 2024 exemplifies ongoing global collaborative efforts toward systematic multi-omics atlas construction across species and disease states. This synthesis underscores a paradigm shift toward AI-enabled, mechanism-driven analyses, ultimately positioning precision neurology as a realizable framework for individualized diagnosis and targeted interventions in complex brain disorders.

Introduction: A paradigm shift in brain research

The brain, as one of the most intricate organs, comprises approximately 86 billion neurons interconnected into sophisticated networks[1, 2]. A profound understanding of its architecture and functional dynamics is imperative, not only for deciphering the substrates of higher-order cognitive processes such as consciousness and memory but also for elucidating the pathophysiological mechanisms underpinning neurological disorders, including Alzheimer's and Parkinson's diseases (PD) and psychiatric disorders. The brain atlas, which maps the brain's architecture, connectivity, and function, serves as a cornerstone for understanding this intricate organ. Traditionally, brain mapping has relied predominantly on macroscopic imaging techniques, such as magnetic resonance imaging (MRI), and static histological descriptions to construct valuable "reference atlases"[3, 4]. However, these atlases largely represent population-level, averaged templates that capture the "commonality" of brains, yet struggle to encapsulate individual variations, developmental dynamics, and the complex pathological essence of diseases.

We are now in the midst of a profound paradigm shift, where brain atlas research is being reshaped by a series of disruptive technologies[5]. Concurrently, its research objectives are evolving from purely descriptive anatomy towards the deep dissection of neurobiological mechanisms and the development of effective intervention strategies[4]. This transformation is primarily manifested in two complementary aspects.

1) Emerging technologies are the core drivers of this revolution. The integration of AI and multi-omics technologies is dismantling the barriers between data and analysis[6, 7]. AI, particularly deep learning, can extract patterns imperceptible to humans from vast amounts of multi-modal brain imaging, genomic, transcriptomic, proteomic, and metabolomic dataComplementing this, cross-species comparative studies and multi-omics atlas construction across model organisms[8-12] provide an evolutionary perspective for dissecting causal relationships between genes, circuits, and behaviors, while accelerating the deciphering of conserved mechanisms underlying brain disorders[13]. Together, these technological advances converge toward the goal of precision medicine, enabling integration of an individual's genetic background, multi-modal atlas data, and clinical phenotypes to predict disease risk, stratify subtypes, and guide personalized therapeutic decisions[14].

2) Research goals have undergone an equally fundamental evolution. The focus has decisively shifted from descriptive studies asking "what it looks like" to mechanistic analyses probing "how it works" and "how it goes awry"[15]. This shift demands the construction of a comprehensive, dynamic healthy brain atlas as a reference baseline, against which the dysregulation of genes, circuits, and networks in complex disorders such as schizophrenia, depression, and Alzheimer's disease (AD) can be precisely characterized. Beyond mechanistic understanding, the ultimate aim of this new paradigm is the translation of these insights into effective clinical interventions. By leveraging key mechanistic nodes identified through basic research, combined with AI-driven biomarker discovery, the field is accelerating the development of novel neuromodulation technologies, drug targets, and small-molecule therapeutics, with the explicit goal of bridging the gap from bench to bedside[16].

This review aims to systematically outline and elaborate on this paradigm shift. We will delve into how AI and multi-omics are empowering the construction of the next generation of brain atlases; analyze how cross-species integration strategies provide unique insights into human brain disease

mechanisms; and envision how atlas-based personalized diagnosis and therapy can move from concept to reality in the era of precision medicine. By summarizing current challenges—such as data standardization, computational resources, and ethical considerations—and future directions, we hope to provide a clear roadmap for researchers in the field, collectively advancing brain science towards a new era capable of profound understanding, precise diagnosis, and effective intervention for brain disorders **(Figure 1)**.

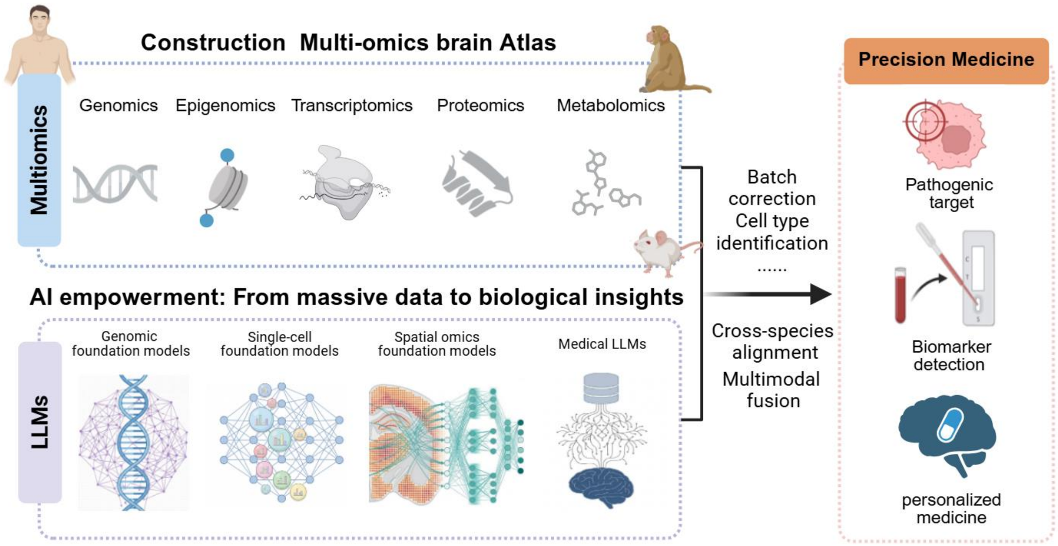

Figure 1: From multi-omics to precision medicine: an AI-enabled brain atlas framework. This figure illustrates the integrative workflow from multi-omics data generation to precision neurology applications. The framework encompasses three major components: (1) Construction of multi-omics brain atlases, where genomics, epigenomics, transcriptomics, proteomics, and metabolomics data are systematically collected across model organisms (human, non-human primates, mouse etc.) to generate comprehensive molecular maps; (2) Advanced AI models, including genomic foundation models, single-cell foundation models, spatial omics foundation models, and medical LLMs, are leveraged to perform batch correction, cell type identification, cross-species alignment, and multimodal data fusion, converting large-scale data into actionable biological knowledge; and (3) Precision medicine applications, where AI-driven analysis enables identification of pathogenic targets, detection of disease-specific biomarkers, and development of personalized therapeutic strategies. This integrated framework exemplifies the paradigm shift from descriptive atlas construction to mechanism-driven precision interventions, ultimately translating multi-dimensional molecular insights into clinically actionable solutions for brain disorders.

Multi-omics mapping technology platform

The breakthrough of neuroscience have historically been contingent upon technological innovation. The development and maturation of single-cell multi-omics technologies have enabled systematic investigation of brain complexity at cellular resolution. These technologies permit simultaneous profiling of transcriptomic, epigenomic, proteomic, and genomic features within individual cells, thereby facilitating the construction of high-resolution molecular atlases of brain cell populations and enhancing our understanding of neuronal diversity and the molecular determinants of functional specialization **(Figure 2)**[8, 17, 18].

Within the single-cell omics technology framework, single-cell RNA sequencing (scRNA-seq) has become a fundamental tool for identifying neuronal and glial cell types. Widespread use of commercial platforms (e.g., 10x Genomics, DNBelab C4, Stereo-cell, SMART-seq) has enabled researchers to establish detailed cellular taxonomies for brain regions across multiple species[8, 17-20]. Concurrently, single-cell chromatin accessibility assays (scATAC-seq/snATAC-seq) provide crucial insights into the epigenetic mechanisms governing brain cell development and differentiation by revealing chromatin openness that dictates cell fate[21, 22]. Single-cell proteomic technologies (e.g., CITE-seq, Chip-Tip) facilitate the detection of cell surface proteins, aiding in the fine subtyping and functional state analysis of immune cells (like microglia) in the central nervous system[23, 24]. Single-cell metabolomics technologies, primarily based on mass spectrometry approaches such as scMEP (single-cell metabolic profiling by epitope-based), enable the characterization of hundreds of metabolites in individual neurons and glial cells, revealing metabolic heterogeneity between cell types and functional states[25, 26]. These technologies have proven particularly valuable for neuroscience research by detecting neurotransmitters, amino acids, lipids, and other small molecules that reflect the biochemical state of individual brain cells, providing insights into neuronal activity, cellular plasticity, and metabolic dysregulation in brain disorders. Furthermore, joint single-cell multi-omics technologies (e.g., SIDR, SPLIT-seq, SHARE-seq), capable of capturing multiple molecular layers from the same cell, provide direct evidence for constructing complete gene regulatory networks, significantly advancing our comprehension of the mechanisms determining brain cell identity[27-31]. Single-cell genomics approaches, utilizing whole genome amplification and high-throughput sequencing, have revealed extensive somatic mutations in individual neurons, including single-nucleotide variants (SNVs), copy number variations, and retrotransposon insertions[32]. These technologies have uncovered that each neuron harbors thousands of unique somatic mutations that accumulate throughout development and aging, serving as endogenous markers for lineage tracing and providing insights into brain development, neuronal diversity, and the pathogenesis of neurological disorders including epilepsy, autism spectrum disorder, and focal cortical dysplasia[32, 33].

However, conventional single-cell techniques inevitably lose the native spatial context of cells during tissue dissociation[34]. Given that the brain is a highly structured organ whose function strictly depends on precise spatiotemporal architecture, this limitation posed a critical challenge, spurring the rapid advancement of spatial omics technologies. High-resolution spatial transcriptomic techniques like Stereo-seq[35] and its enhanced version Stereo-seq V2[36], with their subcellular resolution and whole-transcriptome coverage, allow molecular expression data to be precisely anchored to their original tissue locations. This enables the direct "observation" of distribution patterns for specific cell types and genes across different brain cortices, nuclei, and even fine laminar structures. Similarly, other technologies like 10x Visium, MERFISH, Slide-seq, and STARmap, each with unique strengths in resolution, throughput, and multi-gene detection capability, complement each other and collectively propel the application of spatial transcriptomics in brain research[1, 17, 37-39].

Currently, spatial multi-omics technologies are driving brain science from a singular transcriptomic dimension towards a new phase integrating spatial information for proteins, metabolites, and chromatin. Spatial proteomic technologies (e.g., Stereo-CITE, CODEX, MIBI) enable the simultaneous in-situ detection of dozens of proteins, offering novel perspectives for studying brain cellular spatial organization and cell-cell interactions[40-42]. Spatial metabolomics technologies

(e.g., SEAM) can reveal the distribution of small molecule metabolites in brain tissue, linking metabolic states to functional brain regions[43]. Truly revolutionary joint spatial multi-omics technologies (e.g., DBiT-seq, Spatial-ATAC-RNA-seq, Spatial CITE-seq, SM-Omics) go a step further, allowing researchers to concurrently obtain transcriptomic and epigenomic information from the same tissue section, providing unprecedented possibilities for understanding the epigenetic regulation of gene expression within the native microenvironment[44-48].

The integration of these multi-omics technologies has established a framework for multi-dimensional, high-resolution brain cell atlas construction. These approaches enable systematic investigation of the brain's cellular architecture, molecular regulatory networks, and functional mechanisms, and provide methodological tools for examining brain dynamics across development, aging, and disease states. Continued refinements in resolution, throughput, and multi-modal integration capabilities are advancing efforts toward comprehensive characterization of brain complexity.

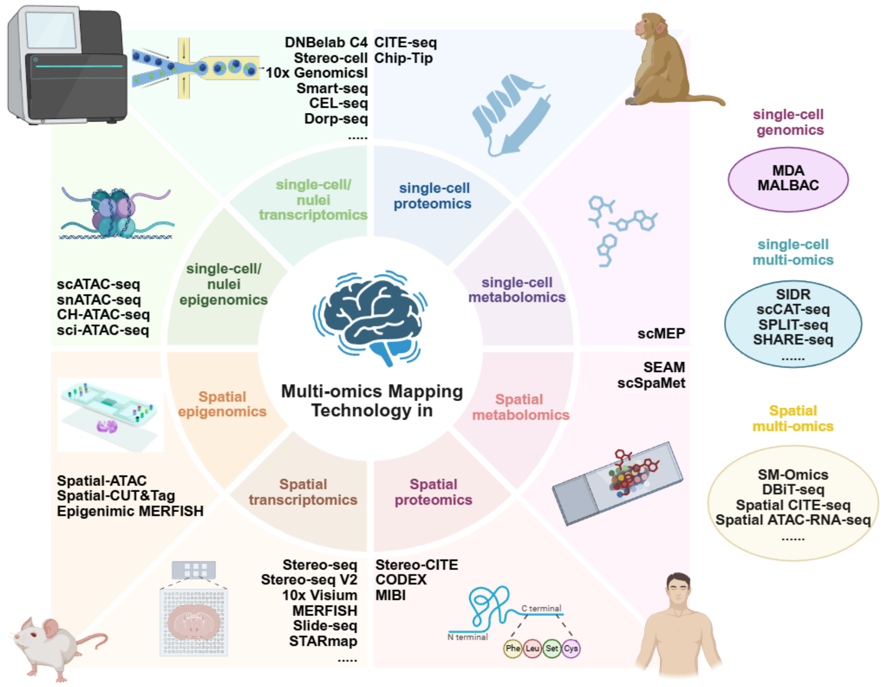

Figure 2: Multi-omics technology in brain science research. This figure illustrates the comprehensive multi-omics technology ecosystem for brain cell atlas construction. Single-cell/nuclei technologies enable molecular profiling of individual cells across transcriptomics (e.g., 10x Genomics, DNBelab C4, Stereo-cell), epigenomics (scATAC-seq, snATAC-seq), proteomics (e.g., CITE-seq, Chip-Tip), metabolomics (e.g., scMEP), and genomics (MDA, MALBAC). Single-cell multi-omics integration platforms (e.g., SIDR, SPLIT-seq, PHAGE-ATAC) simultaneously capture multiple molecular layers from the same cell. Spatial technologies preserve native tissue architecture while mapping molecular information, including spatial transcriptomics (e.g., Stereo-seq, 10x Visium, MERFISH), spatial proteomics (e.g., Stereo-CITE, CODEX, MIBI), spatial epigenomics (e.g., Spatial-ATAC, Epigenimic MERFISH) and spatial metabolomics (e.g., SEAM). Spatial multi-omics platforms (e.g., SM-Omics, Spatial CITE-seq) enable concurrent multi-modal detection with spatial resolution. Model organisms depicted include mouse, non-human primates,

and humans, representing the cross-species comparative framework essential for translational brain research.

Multi-omics brain atlas: A panoramic view from health to disease

The construction of comprehensive brain atlases represents a critical bridge connecting multi-omics technologies to clinical applications in precision neurology . These atlases provide systematic reference frameworks that span the entire spectrum from healthy brain states to diverse pathological conditions, enabling researchers to identify disease-specific cellular and molecular alterations through comparative analysis[49]. This integrated approach encompasses multiple dimensions: it leverages multi-omics profiling across various model organisms to establish robust baseline references [50], tracks dynamic changes throughout developmental and aging trajectories [51], and systematically investigates a broad range of brain disorders including neurodegenerative diseases, psychiatric conditions, and neurodevelopmental disorders [52]**(Figure 3)**. This section examines how multi-omics brain atlases are being systematically constructed for both healthy and diseased states, demonstrating their transformative impact on understanding brain function and dysfunction.

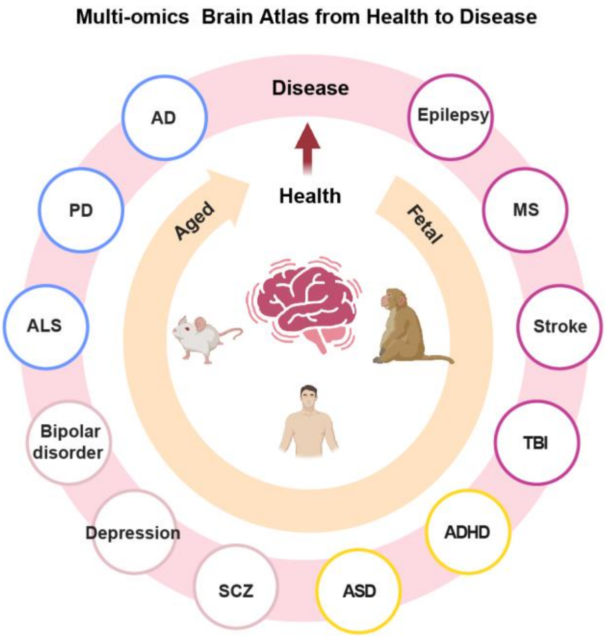

Figure 3: The schematic of multi-omics brain atlas spanning from healthy states to diverse pathological conditions. The central core represents the healthy brain atlas constructed from multi-omics profiling across model organisms (rodents, non-human primates, and humans), serving as the reference foundation. The inner ring depicts temporal dimensions spanning fetal development to aged stages, highlighting dynamic changes throughout the lifespan. The outer ring encompasses brain disorders investigated through atlas-based approaches: neurodegenerative diseases (AD, PD, ALS), psychiatric disorders (depression, SCZ, bipolar disorder), neurodevelopmental disorders (ASD, ADHD), and other neurological conditions (epilepsy, MS, stroke, TBI).

I. Multi-omics cellular mapping of the healthy human brain

The human brain represents a biological system of exceptional structural and functional complexity. Constructing a comprehensive cellular atlas of human brain is pivotal to understanding neural function, developmental processes, aging-related changes, and the pathophysiology of neurological disorders[53]. Recent advances in single-cell sequencing and spatial omics technologies have enabled a transition from region-based neuroanatomical descriptions to cellular-resolution characterization[54, 55]. This shift has facilitated the systematic cataloging of cell types and their molecular profiles, enabling the construction of multi-scale, high-resolution atlases of the healthy human brain.

Large-scale brain cell atlas initiatives, exemplified by the Human Brain Cell Atlas (HBCA), are revolutionizing our understanding of cerebral cellular architecture[56]. By integrating single-cell multi-omics data across regions and development, these projects have unveiled an unprecedented degree of cellular diversity and region-specific specialization[50]. Spatially resolved transcriptomics and computational methods have been pivotal in mapping this complexity, leading to a refined neuronal taxonomy and the discovery of extensive functional heterogeneity among glial cells[54, 57]. Furthermore, cellular-resolution maps of key structures like the hippocampus and cerebellum are directly elucidating the cellular substrates of learning, memory, and motor coordination, moving beyond anatomical description to mechanistic insight[58].

Extending the perspective to developmental timelines, brain atlas research has further uncovered the dynamic blueprint of brain construction. Spatiotemporal atlas studies of the fetal brain have meticulously delineated the molecular programs governing neurogenesis, neuronal migration, and synaptogenesis. Multi-omics analyses of critical developmental windows have provided new insights into the regulatory mechanisms driving the brain's rapid dynamic changes. The precise spatiotemporal regulation of gene expression during development not only shapes a healthy brain but also offers crucial clues for understanding interindividual differences in neuroplasticity and susceptibility to brain disorders[51, 59].

In summary, spanning macroscopic regions to microscopic cells, and static classifications to dynamic development, integrated multi-omics brain atlases are providing a comprehensive view of the brain in health. This continuously expanding and refining blueprint will ultimately serve as the definitive guide for decoding the brain—understanding how it is built, how it functions, and why it fails.

II. Multi-omics cellular mapping of brain disorders

The grand challenge of human brain research is to fundamentally understand its biological essence, with the primary mission of alleviating the burden of brain-related illnesses[5]. Through a synergistic integration of multidisciplinary tools including multi-omics, we are meticulously deconstructing the brain's architecture—from molecular synapses to large-scale circuits—that underlies behavior and cognition[56, 58]. This profound knowledge is the key to developing targeted interventions for disorders ranging from Alzheimer's to depression, while also illuminating the pathways to lifelong brain health[49].

Single-cell multi-omics studies have profoundly advanced our understanding of neurological diseases, especially AD. By simultaneously analyzing multiple layers of information—such as gene expression and epigenetic modifications—at single-cell resolution, the intricate molecular changes

within different brain cell types have profoundly transformed our understanding of AD progression[49, 60-62], offering an unprecedented perspective for understanding disease mechanisms and identifying new therapeutic targets. Multi-omics studies have delineated1 the accumulation of amyloid-β plaques, the spread of Tau pathology, and the dynamic, targetable transition of microglia to a disease-associated state[63-65]. In the effort of systematically research using multi-omics, AD is increasingly viewed as a disorder of cellular networks, where the interplay between proteopathy and glial responses determines outcome, highlighting the therapeutic promise of precisely modulating microglial phenotypes alongside targeting Tau propagation[66, 67]. Beyond AD, single-cell multi-omics studies have expanded to elucidate a broad spectrum of neurological disorders, each with distinct pathological features. For instance, in PD, multi-omics analyses have uncovered the molecular underpinnings of dopaminergic neuron loss in the substantia nigra and the cellular responses surrounding Lewy body formation, revealing dysregulated pathways in protein aggregation and neuroinflammation[52, 68]. In amyotrophic lateral sclerosis (ALS), integrated omics approaches have delineated the degenerative processes in motor neurons and altered neuron-glia interactions, highlighting key genes involved in excitotoxicity and immune activation[69-72]. Epilepsy research has benefited from multi-omics profiling of epileptic foci, which delineates synaptic remodeling[73]. For autism spectrum disorder (ASD), these studies have exposed cortical developmental anomalies and synaptic connectivity alterations, implicating epigenetic modifications and neural circuit dysfunctions[74]. Similarly, intellectual disability and ADHD investigations have mapped cell-type-specific changes in relevant brain regions, uncovering expression variations in genes related to neurodevelopment and neurotransmission[75]. In multiple sclerosis (MS), multi-omics data have detailed the immune cell infiltration patterns in demyelinating lesions, providing insights into autoimmune mechanisms and potential repair processes[76].

Moving to acute brain injuries, multi-omics approaches have been instrumental in decoding the dynamic cellular responses. In stroke, single-cell analyses of the ischemic penumbra have characterized the complex cell reactions and neurorepair mechanisms, including angiogenesis, glial scar formation, and axonal regeneration[77]. For traumatic brain injury (TBI), omics technologies have captured the spatiotemporal dynamics of injury cascades, such as oxidative stress, inflammation, and cell death pathways, offering a holistic view of recovery and degeneration[78].

In the realm of psychiatric disorders, multi-omics studies have begun to unravel the molecular basis of complex conditions. Schizophrenia research has focused on dopaminergic system dysregulation and prefrontal cortical cell alterations, with omics data identifying aberrant gene networks in synaptic function and immune signaling[79, 80]. Depression studies have emphasized impairments in hippocampal neurogenesis and neuroinflammatory features, linking transcriptomic and epigenetic changes to stress responses and treatment outcomes[81]. For bipolar disorder, multi-omics analyses have revealed molecular abnormalities in emotion-regulation brain regions, including alterations in circadian rhythm genes and neurotransmitter systems, paving the way for personalized therapeutic strategies[82].

Collectively, these multi-omics investigations across diverse brain disorders not only deepen our understanding of disease-specific mechanisms but also foster the development of precision medicine approaches. By integrating data from genomics, transcriptomics, and epigenomics at single-cell resolution, we are building a comprehensive atlas of brain pathologies that bridges molecular insights with clinical applications, ultimately advancing our mission to mitigate the global burden of neurological and psychiatric illnesses.

III. Cross-species brain atlas: an evolutionary biology perspective

A critical dimension underlying the validity of these disease atlases, however, is the degree to which findings derived from model organisms can be meaningfully extrapolated to human pathophysiology. Cross-species comparative atlas construction directly addresses this question, providing an evolutionary framework for distinguishing conserved disease mechanisms from species-specific biological features, and for rationally calibrating the translational relevance of non-human model systems. The Brain Initiative Cell Census Network (BICCN) has provided a comprehensive framework for such analyses by generating cellular atlases across human, non-human primates (rhesus macaque and marmoset), and mouse brains[56, 83]. At the foundational level, comparative multi-omics studies reveal striking evolutionary conservation of basic cellular architecture. Most fundamental cell types, including major neuronal and glial cell classes, are highly conserved across mammalian species[84], and certain cell-type-specific molecular markers and signaling pathways exhibit remarkable cross-species consistency [56, 85, 86]. This conservation provides reliable anchor points for translational research and validates the use of animal models for investigating core neurobiological mechanisms. However, layered upon this conserved foundation are significant species-specific elaborations that distinguish the human brain. The most prominent anatomical feature is the substantial expansion of the human neocortex, particularly in prefrontal and temporal association cortices, which correlates with enhanced cognitive capabilities. At the cellular level, single-cell transcriptomic analyses have identified specifically expanded neuronal subtypes in the human cortex , with these cells expressing human-specific genes potentially contributing to higher-order cognitive functions [87, 88]. Notably, the human cortex displays elevated proportions of upper-layer projection neurons and more complex interneuron subtypes[89]. Beyond changes in cellular composition, species-specific differences manifest in cellular proportions, subtype classifications, and gene expression levels[90]. Complementary epigenomic studies have illuminated the regulatory mechanisms underlying these evolutionary innovations. Comparative analyses have identified human-specific regulatory elements enriched near genes related to neural development and synaptic function[91], underscoring the crucial role of gene regulatory evolution—rather than coding sequence changes alone—in shaping human brain evolution[92]. These evolutionary divergences carry important implications for translational neuroscience. Cross-species comparisons have revealed that genes associated with neurodegenerative diseases, such as Alzheimer's disease, are not always expressed in equivalent cell types between humans and mouse[93, 94]. This cellular-level species specificity necessitates cautious interpretation when extrapolating findings from animal models to human pathophysiology, highlighting the importance of integrating human-derived data into preclinical research paradigms.

Mice represent the most commonly used model organism in neuroscience research, with their genomic manipulability and relatively short lifespan making them ideal tools for studying brain development and disease mechanisms[95]. However, cross-species atlas comparisons have revealed important limitations of mouse models[96]. The human brain exhibits substantial differences from mice in size, cortical parcellation, and cellular composition[90], with certain human-specific cell types and gene expression patterns being absent or expressed at very low levels in mice[89]. These differences represent one potential contributor to the frequent failure of therapeutic strategies[97]. In addition, non-human primates, due to their closer phylogenetic relationship to humans, more closely resemble humans in brain structure and cognitive function, and are therefore considered

more ideal models for translational research[98, 99]. Single-cell atlas comparisons demonstrate that humans and non-human primates exhibit high similarity in cell type composition and gene expression patterns, particularly in brain regions associated with higher-order cognition[100]. Nevertheless, even in non-human primates, significant differences from humans persist, including the absence of human-specific genes and variations in certain cell type proportions[88]. Furthermore, non-human primate research faces ethical, financial, and technical challenges that limit its widespread application.

To overcome the limitations of single-species models, multi-species integrative research strategies are emerging. By conducting mechanistic studies and genetic manipulation experiments in mouse models, validating key findings in non-human primate models[101], and integrating human brain tissue and organoid studies[56], researchers can establish a more complete and reliable framework for disease understanding and therapeutic target validation. Cross-species cellular atlases provide an essential reference framework for such integrative research, helping researchers identify conserved and species-specific mechanisms and rationally select and interpret animal model research findings.

AI empowerment: Large Language Foundation Models Revolutionizing Brain Multi-Omics Analysis

The unprecedented complexity of the brain, encompassing hundreds of molecularly distinct cell types[102], intricate spatial organization, and disease-associated genetic variation distributed across vast non-coding regulatory landscapes[103] has long resisted systematic computational interpretation. The emergence of large language foundation models (FMs) trained on biological sequences offers a transformative opportunity: by learning the statistical grammar of DNA, transcriptomes, and proteins from tens of millions of biological sequences, these models derive representations that generalize across tasks, datasets, and species in ways that task-specific models cannot[104]. This section surveys representative FMs across four domains: genomics, single-cell transcriptomics, spatial omics, and clinical medicine, with an emphasis on their architecture, scale, and direct relevance to brain science (**Table 2**).

Table 2. AI Foundation Models Applied in Brain Multi-Omics Research

Model	Author & Time	Task	Brain Science Focus
DNABERT-2	Zhou et al.[105]	DNA	Predicts regulatory elements and noncoding variants across species
HyenaDNA	Nguyen, E., et al.[106]	DNA	Models long-range genomic interactions at single-nucleotide resolution
Genos	Lin, A., et al.[107]	DNA	Analyze the human genomic data of a large population
Evo 2	Brixi, G., et al.[108]	DNA	Predicts pathogenic noncoding variants across eukaryotic genomes
AlphaGenome	Avsec, Ž., et al.[109]	DNA	Predicts gene expression, chromatin accessibility, and regulatory variant effects from DNA sequence
AlphaFold2	Jumper, J., et al.[110]	Protein	Predicts protein 3D structures from sequences; models Aβ42 aggregation in Alzheimer's disease
AlphaFold3	Abramson, J., et al.[111]	Protein	Predicts biomolecular complex structures; characterizes structural effects of AD-associated missense variants
ESM-2	Frank, M., et al.[112]	Protein	Predicts protein structure and amyloid aggregation propensity of tau, APP, and α-synuclein
AlphaMissense	Cheng, J., et al.[113]	Protein	Classifies missense variant pathogenicity across neurological disease genes (LRRK2, SNCA, APP, PSEN1)
AlphaPeptDeep	Zeng, W.F., et al.[114]	Protein	Predicts peptide RT, CCS, and MS2 intensities for PTM-rich brain proteome DIA analysis
scGPT	Cui, H., et al.[115]	scRNA	Annotates cell types and integrates batches from single-cell brain transcriptomics data
Geneformer	Theodoris, C.V., et al.[116]	scRNA	Predicts gene dosage sensitivity and network biology in fetal brain cells
scFoundation	Hao, M., et al.[117]	scRNA	Predicts cell-type-specific drug responses across CNS cell populations
UCE	Rosen, Y., et al.[118]	scRNA	Annotates cell types across species without fine-tuning using universal cell embeddings
GeneCompass	Yang, X., et al.[119]	scRNA	Annotates mouse brain cell types with knowledge-guided gene regulatory integration
CellFM	Zeng, Y., et al.[120]	scRNA	Annotates rare brain cell populations including disease-associated microglia and interneuron subtypes
CAPTAIN	Ji, B., et al.[121]	scRNA	Models joint RNA–protein representations and intercellular dynamics in neural microenvironments
Nicheformer	Tejada-Lapuerta, A., et al.[122]	Spatial	Characterizes tissue niches and brain region identity from spatial transcriptomics data
Novae	Blampey, Q., et al.[123]	Spatial	Identifies spatial domains and parcellates brain regions across unseen gene panels
OmiCLIP	Chen, W., et al.[124]	Spatial	Predicts spatial transcriptomics from H&E histology images for brain cell-type decomposition
scGPT-spatial	Wang, C., et al.[125]	Spatial	Decodes, imputes, and deconvolves spatial gene expression across brain tissue sections
OmniCell	Pang, J., et al.[126]	Spatial	Models intra- and inter-cellular spatial dependencies in aging mouse brain atlas
Med-PaLM 2 & Med-PaLM M	Singhal, K., et al.[127]	Medical LLM	Answers medical questions and analyzes multimodal neurological cases at expert level

Model	Author & Time	Task	Brain Science Focus
GatorTron	Yang, X., et al.[128]	Medical LLM	Extracts psychiatric and neurological information from unstructured clinical notes
GatorTronGPT	Peng, C., et al.[129]	Medical LLM	Generates synthetic clinical text and supports psychiatric decision-making
Meditron	Chen, Z., et al.[130]	Medical LLM	Predicts ICU mortality for patients with mental disorders from clinical notes

I. Genomic Foundation Models

More than 90% of neurological disease-associated variants identified by GWAS reside in non-coding regulatory regions, yet the functional interpretation of these variants has remained a bottleneck. Genomic foundation models (gFMs) address this by learning the regulatory grammar encoded in DNA sequence, evolving from transformer-based designs toward hybrid architectures capable of processing megabase-scale contexts at single-nucleotide resolution.

DNABERT-2[105] introduced byte pair encoding (BPE) to replace the k-mer tokenization of its predecessor, dramatically reducing computational cost while achieving competitive performance across genomic classification tasks. Its ability to generalize zero-shot to sequences far longer than those seen during training makes it particularly applicable to the large, complex regulatory domains that govern brain-specific gene expression programs. While DNABERT-2 extended breadth of genomic representation, HyenaDNA extended depth of context. By replacing the transformer's attention mechanism with Hyena operators, HyenaDNA achieves context lengths of up to 1 million base pairs with sub-quadratic scaling[106] — a decisive advance for neuroscience, where neuronal enhancers and silencers can reside hundreds of kilobases from their target genes. This capacity to model distal regulatory interactions is essential for decoding the cell-type-specific gene programs of neurons and glia whose dysregulation underlies much of neurological disease. Building on this foundation of scale and context, Genos pushes genomic modeling to a new tier of population diversity and clinical resolution. As one of the largest gFMs to date, Genos employs a Mixture-of-Experts transformer with 10.27 billion parameters and 1 megabase context, trained on 636 telomere-to-telomere human genome assemblies spanning diverse global populations[107]. The model achieves 93% AUC on pathogenicity prediction, and has demonstrated direct neurological application in variant effect scoring for LRRK2 — the gene most commonly mutated in familial Parkinson's disease — exemplifying how population-scale genomic modeling can be grounded in cell-type-specific neuropathology.

The Arc Institute's Evo series extends this logic across the full diversity of life. The first-generation Evo employs a 7-billion-parameter StripedHyena architecture trained on the OpenGenome dataset spanning bacteria, archaea, and bacteriophages, enabling zero-shot gene essentiality prediction and de novo multi-gene circuit generation at single-nucleotide resolution[131]. Its successor, Evo 2, substantially expands scale and biological scope: 7B and 40B parameter variants trained on approximately 9.3 trillion DNA tokens from all domains of life, with 1 million token context windows and a hybrid Transformer–StripedHyena architecture [108]. Critically, Evo 2 extends to eukaryotic genomes including human, enabling prediction of pathogenic noncoding mutations and clinically relevant variant effects. For brain research, this cross-domain generalization is particularly significant: regulatory variants driving neurological disease often reside in non-coding sequence

elements whose evolutionary conservation and functional logic become interpretable only in the context of deep evolutionary diversity. Whereas the preceding models focus on variant interpretation within linear sequence context, AlphaGenome, developed by Google DeepMind, integrates the outputs of genomic regulation into a unified multi-modal prediction framework. Accepting up to 1 megabase of DNA, AlphaGenome simultaneously predicts across eight functional genomic modalities: gene expression, transcription initiation, chromatin accessibility, histone modifications, transcription factor binding, chromatin contact maps, splice site usage, and splice junction strength, at single-base-pair resolution[109]. Trained on human and mouse genomes, the model matches or exceeds specialist models in 25 of 26 variant effect benchmarks. For neurological disease, its simultaneous multi-modal scoring of regulatory variants enables the identification of a single non-coding variant that disrupts a CTCF binding site, alters neuronal chromatin accessibility, and reduces expression of a disease-relevant gene; this represents a qualitative advance over single-modality interpretation.

Together, these models form a progression from accurate sequence representation, through long-range regulatory context, to population-scale variant interpretation, to integrated multi-modal functional prediction. Collectively, they provide tools to decode the non-coding regulatory landscape that governs brain development and disease.

II. Protein Foundation Models

Proteins are the primary effectors of neurological function and disease: the misfolding of tau, amyloid-beta, and alpha-synuclein drives neurodegeneration, while the structural integrity of synaptic receptors, ion channels, and brain-resident immune proteins determines circuit function and inflammatory state. Protein foundation models have transformed the field by learning the evolutionary and structural grammar of protein sequences from hundreds of millions of examples, enabling structure prediction, variant effect analysis, and peptide property prediction at scales previously inaccessible to experimental methods.

AlphaFold2 and AlphaFold3, developed by Google DeepMind, represent successive breakthroughs in structural biology with direct relevance to neurological disease. AlphaFold2 achieved atomic-accuracy prediction of protein 3D structures from sequence alone[110], winning CASP14 and earning the 2024 Nobel Prize in Chemistry; its open-source structural database has enabled mechanistic studies of neurodegeneration, including prediction of the Aβ42 monomer-to-hexamer aggregation pathway in Alzheimer's disease[132]. AlphaFold3 extended this framework to unified prediction of biomolecular complexes through an integrated diffusion-based architecture[111], and has been applied to characterize structural consequences of seven AD-associated missense pQTL variants across microglial proteins including TREM2, CD33, and PILRB. Where AlphaFold models predict static structures, ESM-2 and ESMFold learn the evolutionary language of protein sequences directly from 250 million sequences spanning 86 billion amino acids, enabling structure prediction and variant effect scoring at 60× the speed of AlphaFold2[133]. For neurodegeneration, ESM-2 embeddings have been applied to predict aggregation propensity and phase separation behavior of tau, APP, and alpha-synuclein[112]. Complementing this, AlphaMissense classified 71 million possible human missense variants by combining AlphaFold2 structural representations with evolutionary constraint signals[113], providing systematic pathogenicity scores across neurodegeneration-associated genes including LRRK2, SNCA, APP, and PSEN1. A distinct class of protein foundation models addresses quantitative brain proteomics. Prosit predicts peptide fragment

ion intensities and retention times, enabling DIA workflows with more than tenfold reductions in false discovery rate and substantially deeper proteome coverage in brain tissue studies[134]. AlphaPeptDeep extends this to simultaneous prediction of retention time, collisional cross section, and MS2 spectra, with transfer learning enabling rapid adaptation to novel PTMs critical for studying synaptic protein phosphorylation and ubiquitination in disease[114]. DeepLC complements both by providing zero-shot retention time prediction for PTMs absent from its training data, enabling unbiased discovery of novel modification sites on neurological disease proteins[135].

Together, these models form a coherent pipeline from atomic structure prediction and variant pathogenicity classification to deep quantitative proteome profiling — collectively decoding neurological disease mechanisms at the protein level.

III. Single-Cell Foundation Models

Understanding the cellular diversity of the human brain requires more than cataloguing gene expression: it demands representations that can annotate hundreds of cell types, predict responses to perturbations, and generalize across datasets, species, and disease states. Single-cell foundation models (scFMs) address this challenge by treating genes as tokens and cells as sentences, learning context-dependent gene interaction patterns from millions of transcriptomes through self-supervised pretraining.

scGPT pioneered the GPT-style generative architecture for single-cell data, pretraining on over 33 million cells across 441 studies and 51 human organs[115]. Fine-tuned on Human Brain Cell Atlas data for perirhinal cortex annotation, scGPT demonstrated that general-purpose pretraining could produce representations directly applicable to the specialized cellular taxonomy of the brain. Its batch integration capabilities provide a practical tool for harmonizing the multi-site transcriptomic datasets that characterize large-scale brain atlas efforts. While scGPT established the generative paradigm, Geneformer extended its interpretive power through in silico perturbation. Employing BERT-style masked language modeling, Geneformer’s key contribution to neuroscience lies in its capacity to predict the transcriptional consequences of silencing or overexpressing specific genes in silico, without the need for corresponding experimental data[116]. This capability has been applied to dosage sensitivity analysis of disease-associated genes in neurons and fetal cerebrum, contexts where generating matched perturbation data is both technically demanding and ethically constrained. Complementing Geneformer's perturbation modeling, scFoundation brought architectural innovation to the challenge of expression resolution. Its asymmetric transformer with 100 million parameters and direct value projection preserves full expression magnitudes rather than binarized rank data, enabling prediction of cell-type-specific drug responses with high AUPR[117]. For brain pharmacology, this offers a computational route to model CNS drug effects across the heterogeneous cellular landscape of the human brain — a critical capability given the difficulty of obtaining matched human brain tissue for drug testing. Building on this cross-species capability, GeneCompass introduces knowledge-informed pretraining that integrates gene regulatory networks, promoter sequences, and transcription factor–target relationships alongside expression data. Trained on 120 million cells from human and mouse, GeneCompass demonstrates superior cross-species cell type annotation on brain datasets[119] — its incorporation of regulatory logic rather than expression alone offering a natural fit for neuroscience, where transcription factor programs define and maintain cell identity throughout life and disease. CellFM, currently the largest scFM with 800

million parameters trained on 100 million human cells using a modified RetNet framework, exemplifies how scale directly translates to brain research utility[120]. Its outperformance on cell annotation, perturbation prediction, and gene function prediction extends specifically to the rare brain cell populations — interneuron subtypes, specialized astrocytes, disease-associated microglia — that are underrepresented in smaller training corpora but are often the most biologically and clinically informative in neurological disease.

Across these models, a clear developmental arc emerges: from pioneering generative architecture, through mechanistic perturbation modeling, pharmacological prediction, cross-species generalization, regulatory knowledge integration, and finally the scale required to represent the full complexity of brain cellular diversity.

IV. Spatial Omics Foundation Models

Dissociation-based single-cell methods sacrifice a fundamental dimension of brain organization: tissue architecture. The laminar structure of the cortex, the trisynaptic circuit of the hippocampus, and the perivascular niches of brain-resident immune cells: these spatial relationships are not merely anatomical decorations but are mechanistically constitutive of brain function. Spatial omics foundation models restore this context, learning representations that integrate transcriptional identity with tissue topology.

Nicheformer was the first foundation model to train jointly on dissociated single-cell and spatially resolved transcriptomics data, pretraining on SpatialCorpus-110M, over 57 million dissociated and 53 million spatially resolved cells across 73 tissues[122]. By demonstrating that models trained solely on dissociated data systematically fail to recover the complexity of tissue microenvironments, Nicheformer established a key principle for brain atlas construction: spatial context is not optional. Its zero-shot transfer across spatial platforms makes it immediately applicable to the heterogeneous landscape of brain spatial transcriptomic datasets. Extending spatial generalization further, Novae introduced a graph neural network-based architecture trained on approximately 30 million spatial transcriptomics cells, with the distinctive capability of zero-shot domain inference across unseen gene panels and technologies[123]. Validated against the Allen Reference Atlas on mouse brain sections, Novae accurately recovers hierarchical neuroanatomical boundaries, a technology-agnostic parcellation capability essential for integrating brain datasets generated on different spatial platforms, which currently remain difficult to compare directly. Where Novae parcellates based on transcriptional domains, OmiCLIP bridges modalities: by pioneering contrastive learning between H&E histology images and spatial transcriptomic profiles across 2.2 million paired samples from 32 organs, OmiCLIP enables prediction of molecular spatial organization directly from tissue morphology[124]. For neuroscience, this capability is transformative: it makes molecular-resolution spatial analysis accessible to the vast archives of neuropathology tissue, including historical samples from AD, PD, and other neurological diseases that predate spatial sequencing technologies by decades.

scGPT-spatial and OmniCell represent the current frontier of spatial modeling, tackling the practical demands of multi-platform brain atlas construction from complementary directions. scGPT-spatial extends the scGPT framework through continual pretraining on SpatialHuman30M (30 million profiles from Visium, Visium HD, MERFISH, and Xenium platforms across 821 tissue slides), with a Mixture-of-Experts decoder that enables protocol-aware gene expression decoding[125]. OmniCell takes a structurally distinct approach: as the first model to jointly represent intra-cellular

gene expression and inter-cellular spatial dependencies within a unified architecture, it serializes spatially adjacent cells as context during training, learning representations that simultaneously encode transcriptional state and tissue topology[126]. Benchmarked on a MERFISH mouse brain aging atlas spanning 31 datasets, OmniCell outperforms scGPT-spatial and Nicheformer on spatial clustering and achieves rare cell detection accuracy 13 percentage points above competing models, a margin with direct implications for detecting the sparse, spatially restricted cell populations most relevant to neurological disease.

V. Medical LLMs: Accelerating Neurological Disease Diagnosis

The translation of molecular insights into clinical benefit requires bridging the gap between biological complexity and medical reasoning. Medical large language models have achieved this at scale, reaching clinician-level performance on standardized medical examination benchmarks and enabling new approaches to diagnosis, drug repurposing, and clinical text analysis in neurological disease.

Med-PaLM2 from Google was the first AI system to achieve expert-level performance on MedQA[127], with 86.5% accuracy and responses preferred over physician answers on 8 of 9 clinical evaluation axes. Its multimodal successor, Med-PaLM M, integrates imaging, clinical text, and genomic data in a unified framework, a capability directly applicable to the multimodal nature of neurological case analysis, where diagnosis typically depends on the synthesis of MRI findings, clinical presentation, biomarker profiles, and genetic information. GatorTron, developed by the University of Florida and NVIDIA, scales clinical language modeling to its current frontier: 8.9 billion parameters trained on over 90 billion words, including 82 billion words of de-identified clinical notes[128]. Its generative successor GatorTronGPT extends to 20 billion parameters and demonstrates 9.6% improvement on natural language inference tasks. For neurology, GatorTron's deep grounding in clinical documentation enables extraction of structured neurological information — symptom timelines, medication responses, disease progression markers — from the unstructured free text that constitutes the majority of clinical knowledge. Complementing these institutional systems, Meditron from EPFL and Yale provides the leading open-source alternative: a Llama-2-based model (7B and 70B versions) achieving 77.6% accuracy on MedQA, within 1% of GPT-4, trained on the GAP-Replay corpus of 48.1 billion tokens including 46,000 clinical practice guidelines. Meditron's open availability makes it a critical resource for academic neurology research, where access to proprietary clinical LLMs is constrained.

The clinical impact of these models is already materializing in neurological disease. LLM-driven drug repurposing analyses, validated on real-world data from the Vanderbilt and All of Us cohorts, have identified metformin, simvastatin, and losartan as associated with reduced Alzheimer's disease risk[136]. Multi-source LLM integration achieves 0.849 F1 for glioblastoma presence classification and 0.929 for tumor stability assessment from radiology reports. At the regulatory frontier, the FDA has cleared AI-powered neurology devices including icobrain aria — the first AI software to detect and grade amyloid-related imaging abnormalities (ARIA) critical for monitoring anti-amyloid therapies such as lecanemab — alongside NeuroQuant 5.0, which provides deep learning-based brain segmentation with ARIA detection for Alzheimer's monitoring. These approvals signal a shift from research demonstration to clinical deployment, marking the beginning of AI-assisted neurological care at scale.

VI. Convergence Toward Multimodal Integration

The most significant trend is convergence toward multimodal integration. Models like Genos pair 10B parameter genomic encoders with 4B parameter language models for omics-text reasoning. UCE leverages ESM-2 protein embeddings to enable cross-species cell type annotation. Nicheformer jointly trains on dissociated and spatial data with organism and assay tokens enabling transfer across modalities. CAPTAIN[121] exemplifies a new generation of multimodal single-cell foundation models pretrained on over four million cells with concurrently measured transcriptomes and a curated repertoire of 382 surface proteins, learning unified representations by explicitly modeling cross-modality dependencies between RNA and protein; the model uncovers protein-driven intercellular dynamics including immune interaction patterns linked to COVID-19 severity. Similarly, OmniCell unifies scRNA-seq and spatial transcriptomics by serializing spatial neighborhood graphs within a shared Transformer architecture pretrained on 67 million cells, enabling seamless transfer across modalities and platforms. At the genomic level, AlphaGenome integrates predictions across eight functional modalities (gene expression, splicing, chromatin accessibility, histone modifications, transcription factor binding, chromatin contacts, and splice junction coordinates) from a single unified model, enabling multi-dimensional scoring of regulatory variants relevant to neurological disease. For brain multi-omics specifically, these capabilities translate to unprecedented analytical power. Genomic foundation models predict variant effects in neurological disease genes with >90% accuracy. Single-cell models trained on 100+ million cells enable automated brain cell type annotation and perturbation prediction. Spatial models preserve the three-dimensional organization critical to understanding neural circuits. Medical LLMs synthesize clinical literature and patient records for diagnosis and treatment optimization.

The path to clinical translation is accelerating. Models like Genos and Meditron are released under open-source licenses enabling academic validation. FDA clearances for brain-specific AI tools have reached approximately 30 devices. The integration of foundation model predictions with established brain atlases from Allen Brain Institute, Human Cell Atlas, and the BRAIN Initiative Cell Atlas Network (BICAN) provides the validation infrastructure necessary for clinical deployment. As these models continue scaling, with the largest now exceeding 40 billion parameters and training datasets approaching 10 trillion bases, their emergent capabilities will increasingly define the frontier of computational neuroscience and precision neurology.

Clinical applications of AI-driven multi-omics integration in neurological diseases

The sophisticated AI methodologies described above provide the essential computational infrastructure for addressing one of the most pressing challenges in precision neurology: the accurate stratification of clinically heterogeneous brain disorders into biologically meaningful subtypes. Traditional diagnostic frameworks, predominantly reliant on clinical symptomatology and gross imaging features, frequently fail to capture the underlying molecular heterogeneity that drives variable disease trajectories and differential treatment responses. The integration of AI-driven multi-omics analysis fundamentally transforms this paradigm by enabling data-driven disease reclassification based on comprehensive molecular signatures rather than phenomenological observations alone.

Specifically, machine learning classifiers based on single-cell atlases can identify disease-specific cell subpopulations and molecular biomarkers from patient cerebrospinal fluid or peripheral blood

samples[137]. Research focused on AD has developed classification models based on single-cell transcriptomic data, whereby analysis of gene expression patterns in peripheral blood mononuclear cells enables differentiation between AD patients and healthy controls with considerable accuracy[137, 138]. Regarding prognostic prediction, AI models can extract prognosis-related features from patient multi-omics data to forecast disease progression rates and treatment responses. Research on glioblastoma has utilized single-cell transcriptomic data to identify tumor cell subpopulations associated with poor prognosis[139, 140], with these cells exhibiting stem cell-like characteristics and elevated expression of therapy resistance-associated genes. Predictive models based on the abundance of these cell subpopulations can accurately forecast patient survival, providing evidence for individualized treatment decisions. Time-series deep learning models, including recurrent neural networks and Transformer architectures, have been applied to analyze longitudinal multi-omics data, capturing the dynamic evolution of disease and predicting disease trajectories.

In drug target discovery, AI-driven multi-omics analysis enables systematic identification of key disease drivers and potential therapeutic targets[141, 142]. Gene regulatory network inference methodologies[143] identify aberrantly activated transcription factors and signaling pathways in disease states, with these molecules representing potential therapeutic targets. Cell-cell communication analysis has revealed abnormal ligand-receptor interactions within the disease microenvironment[144], providing insights for therapeutic strategies targeting intercellular communication. Deep learning-based drug repositioning approaches integrate multi-omics data with drug-target interaction databases to predict the potential efficacy of existing drugs against brain disorders, thereby accelerating drug development processes. Implementation of precision medicine requires understanding inter-patient heterogeneity and formulating individualized treatment regimens. Single-cell multi-omics profiling enables molecular stratification of patients into distinct disease subtypes with differential treatment responses[145, 146]. For instance, in glioblastoma, integration of single-cell transcriptomics and chromatin accessibility data has identified patient subgroups characterized by distinct cellular compositions and regulatory landscapes, which correlate with differential responses to immunotherapy and targeted therapies[145, 147]. Machine learning models trained on patient-specific multi-omics signatures can predict individual responses to therapeutic interventions, enabling clinicians to prioritize treatment options with the highest likelihood of efficacy while minimizing adverse effects. Furthermore, longitudinal monitoring of circulating biomarkers through liquid biopsy approaches, coupled with AI-driven trajectory analysis, facilitates real-time assessment of treatment efficacy and early detection of therapeutic resistance , thereby enabling dynamic treatment adjustments in clinical practice[145, 146].

From laboratory to clinical: challenges and perspectives

Despite the considerable potential demonstrated by multi-omics AI technologies in brain research, the translation from laboratory investigation to clinical application continues to face multiple challenges.

The foremost challenge concerns data quality and standardization. Single-cell and spatial transcriptomic data exhibit substantial technical batch effects[148, 149], rendering data generated across different platforms and laboratories difficult to compare directly[150]. Establishing standardized protocols for data acquisition, processing, and quality control constitutes the foundation for enabling data sharing and cross-study comparisons. International collaborative

initiatives such as the Human Cell Atlas[151] and the Brain Initiative are actively promoting data standardization and open sharing practices, thereby establishing a foundation for field advancement. Besides, clinical validation and regulatory approval represent the final critical steps in technology translation. AI diagnostic and prognostic tools require validation of their efficacy and safety through large-scale, multicenter clinical studies[152], demanding substantial investments of time and resources. Regulatory agencies such as the Food and Drug Administration are establishing approval frameworks for AI medical devices, although specific standards for multi-omics AI tools remain under development. Strengthening collaborations across academia, industry, healthcare, and research institutions, and establishing clinical validation platforms and translational research centers, represent effective strategies for accelerating clinical translation of technologies. As well, the interpretability and trustworthiness of AI models constitute critical requirements for clinical implementation. Explainable AI methodologies, including attention visualization, feature importance analysis, and causal inference techniques, are under active development, aiming to elucidate the biological foundations underlying model decisions. The development of AI architectures with inherent interpretability, such as graph neural networks informed by biological prior knowledge and symbolic reasoning systems, represents an important direction for enhancing model trustworthiness [153]

Notwithstanding above challenges, the field has achieved important breakthroughs. Multiple AI - based medical imaging diagnostic systems have received regulatory approval and been implemented in clinical practice, establishing precedents for brain disease AI diagnostics. The cost of single-cell sequencing continues to decline, and commercial platforms are becoming increasingly mature[154], enabling multi-omics technologies to gradually transition from research tools to clinical diagnostic applications. Several early diagnostic products for brain disease based on liquid biopsy and multi-omics biomarkers are currently in clinical trial phases[155, 156], demonstrating promising diagnostic performance. With continued technological advancement, establishment of standards, and refinement of regulatory frameworks, multi-omics AI technologies are positioned to achieve broader clinical implementation within the next five to ten years.

Looking ahead: The next decade of brain disease research

I. Technological frontiers and future directions

Over the next decade, brain science research is positioned to achieve breakthrough advances across technological, theoretical, and applied dimensions. At the technological level, single-cell multi-omics methodologies will advance toward higher throughput, reduced costs, and more comprehensive molecular coverage. Spatial multi-omics technologies are expected to achieve genuine single-cell and even subcellular resolution, enabling simultaneous detection of tens of thousands of genes, proteins, and metabolites. Advances in live imaging technologies will enable researchers to observe brain activity and molecular dynamics in real time without tissue damage, while integration with optogenetic and chemogenetic approaches will facilitate precise neural circuit manipulation.

In the domain of AI methodologies, the concepts of foundation models and large language models are expanding into the biomedical field with remarkable momentum. Recent years have witnessed the emergence of specialized foundation models for biological sequence analysis, including scGPT[115], Geneformer[116], etc. These general-purpose brain science models pretrained on

massive multi-omics datasets can be applied to diverse downstream tasks through transfer learning, including cell type identification, disease diagnosis, and drug response prediction, substantially reducing the data requirements and training costs associated with specific applications. This convergence of large-scale pretraining, transfer learning, and hybrid reasoning architectures positions AI-driven multi-omics analysis at the forefront of precision neurology advancement.

II. The establishment of the brain project international grand science alliance

Recognizing the complexity and global nature of brain science research, international brain initiative collaborations have continued to deepen. National-level brain science projects such as the U.S. BICCN (BRAIN Initiative Cell Census Network)[56, 157] , the European Union's Human Brain Project[158], China Brain Project[159], and Japan's Brain/MINDS[160] have been underway for many years and have achieved important results . In October 2024, the International Consortium for Primate Brain Mapping (ICPBM) was officially established in Shanghai by Chinese Academy of Sciences, BGI-research group, and the University of Hainan, marking a new phase of global collaboration in brain science research. As one of the organizers, BGI research group provides the up-to-date multi-single cell sequencing and spatial transcriptomic technologies, as well as AI computing power and resources. So far, ICPBM brings together over one hundred scientists from twenty-five countries, with plans to systematically map the multi-omics atlases of marmoset, macaque, and human brains over a 25-year period. This comprehensive mapping will encompass complete cell type classifications, transcriptome-based gene expression patterns, and the projection connectivity of each cell. *Science* magazine published a featured report on this initiative, noting that the scale of the proposed plan is impressive and that it "will have enormous impact on the field". The consortium chair, Professor Mu-ming Poo, emphasized that this initiative will provide crucial insights into the cellular and molecular mechanisms of neurological diseases ranging from stroke to Alzheimer's disease through promoting data sharing, standard unification, resource integration, and collaborative research, pointing the way toward new therapeutic approaches. Looking forward, with technological advances, AI development, and deepening global cooperation, the next decade will witness revolutionary breakthroughs in brain science research. Just as the Human Genome Project ushered in the era of genomic medicine, the brain cell atlas projects such as ICPBM are now opening a new era of precision neurology. This represents both an exciting journey of scientific exploration and a profoundly significant endeavor for human health.

Abbreviations

AI: Artificial Intelligence; AD: Alzheimer's Disease; ADHD: Attention Deficit Hyperactivity Disorder; ALS: Amyotrophic Lateral Sclerosis; ATAC-seq: Assay for Transposase-Accessible Chromatin using Sequencing; BICCN: Brain Initiative Cell Census Network; CITE-seq: Cellular Indexing of Transcriptomes and Epitopes by Sequencing; CODEX: CO-Detection by indEXing; DBiT-seq: Deterministic Barcoding in Tissue for spatial omics sequencing; FDA: Food and Drug Administration; ICPBM: International Consortium for Primate Brain Mapping; MS: Multiple Sclerosis; PD: Parkinson's Disease; SCZ: Schizophrenia; SHARE-seq: Simultaneous High-throughput Analysis of Regulatory Elements and Gene Expression by Sequencing; scRNA-seq: Single-Cell RNA Sequencing; snATAC-seq: Single-Nucleus Assay for Transposase-Accessible Chromatin using Sequencing; TBI: Traumatic Brain Injury.

Funding

This work was supported by National Key Research and Development (R&D) Program of China (2021YFA0805100), Key Program of the National Natural Science Foundation of China (32530027), the National Science and Technology Innovation 2030 Major Program (2021ZD0204400).

Data Availability

No data are associated with this article.

Competing Interests

The authors declare that they have no competing interests

References

1. Fang, R., et al., *Conservation and divergence of cortical cell organization in human and mouse revealed by MERFISH.* Science, 2022. **377**(6601): p. 56-62.
2. Herculano-Houzel, S., *The human brain in numbers: a linearly scaled-up primate brain.* Front Hum Neurosci, 2009. **3**: p. 31.
3. Amunts, K., et al., *BigBrain: an ultrahigh-resolution 3D human brain model.* Science, 2013. **340**(6139): p. 1472-5.
4. Elam, J.S., et al., *The Human Connectome Project: A retrospective.* Neuroimage, 2021. **244**: p. 118543.
5. Jorgenson, L.A., et al., *The BRAIN Initiative: developing technology to catalyse neuroscience discovery.* Philos Trans R Soc Lond B Biol Sci, 2015. **370**(1668).
6. Nam, Y., et al., *Harnessing Artificial Intelligence in Multimodal Omics Data Integration: Paving the Path for the Next Frontier in Precision Medicine.* Annu Rev Biomed Data Sci, 2024. **7**(1): p. 225-250.
7. Kant, S., Deepika, and S. Roy, *Integrative Multi-Omics and Artificial Intelligence: A New Paradigm for Systems Biology.* Omics, 2025. **29**(12): p. 576-587.
8. Chen, A., et al., *Single-cell spatial transcriptome reveals cell-type organization in the macaque cortex.* Cell, 2023. **186**(17): p. 3726-3743 e24.
9. Feng, Z., et al., *A mouse brain stereotaxic topographic atlas with isotropic 1-mum resolution.* Nature, 2025. **645**(8080): p. 448-456.
10. Han, L., et al., *Single-cell spatial transcriptomic atlas of the whole mouse brain.* Neuron, 2025. **113**(13): p. 2141-2160 e9.
11. Toh, H.S.Y., et al., *BrainSTEM: A single-cell multiresolution fetal brain atlas reveals transcriptomic fidelity of human midbrain cultures.* Sci Adv, 2025. **11**(44): p. eadu7944.
12. Zhu, X., et al., *An anatomical and connectivity atlas of the marmoset cerebellum.* Cell Rep, 2023. **42**(5): p. 112480.
13. Zemke, N.R., et al., *Conserved and divergent gene regulatory programs of the mammalian neocortex.* Nature, 2023. **624**(7991): p. 390-402.
14. Abdelaziz, E.H., et al., *Multi-omics data integration and analysis pipeline for precision medicine: Systematic review.* Comput Biol Chem, 2024. **113**: p. 108254.
15. Cherubini, E., et al., *Editorial: Paradigm shifts and innovations in cellular neuroscience.* Front Cell Neurosci, 2025. **19**: p. 1644329.
16. Borchert, R.J., et al., *Artificial intelligence for diagnostic and prognostic neuroimaging in dementia: A systematic review.* Alzheimers Dement, 2023. **19**(12): p. 5885-5904.

17. Qian, X., et al., *Spatial transcriptomics reveals human cortical layer and area specification.* Nature, 2025. **644**(8075): p. 153-163.
18. Chen, D., et al., *Genomic evolution reshapes cell-type diversification in the amniote brain.* Dev Cell, 2025. **60**(13): p. 1900-1915 e5.
19. Liao, S., et al., *Stereo-cell: Spatial enhanced-resolution single-cell sequencing with high-density DNA nanoball-patterned arrays.* Science, 2025. **389**(6762): p. eadr0475.
20. Li, Q., et al., *A single-cell transcriptomic atlas tracking the neural basis of division of labour in an ant superorganism.* Nat Ecol Evol, 2022. **6**(8): p. 1191-1204.
21. Zhong, S., et al., *Decoding the development of the human hippocampus.* Nature, 2020. **577**(7791): p. 531-536.
22. Zhao, Z., et al., *Author Correction: Evolutionarily conservative and non-conservative regulatory networks during primate interneuron development revealed by single-cell RNA and ATAC sequencing.* Cell Res, 2023. **33**(7): p. 569-573.
23. Ye, Z., et al., *Enhanced sensitivity and scalability with a Chip-Tip workflow enables deep single-cell proteomics.* Nat Methods, 2025. **22**(3): p. 499-509.
24. Stoeckius, M., et al., *Simultaneous epitope and transcriptome measurement in single cells.* Nat Methods, 2017. **14**(9): p. 865-868.
25. Qi, M., et al., *Single Cell Neurometabolomics.* ACS Chem Neurosci, 2018. **9**(1): p. 40-50.
26. Nemes, P., et al., *Single-cell metabolomics: changes in the metabolome of freshly isolated and cultured neurons.* ACS Chem Neurosci, 2012. **3**(10): p. 782-92.
27. Ma, S., et al., *Chromatin Potential Identified by Shared Single-Cell Profiling of RNA and Chromatin.* Cell, 2020. **183**(4): p. 1103-1116 e20.
28. Li, Y., et al., *UDA-seq: universal droplet microfluidics-based combinatorial indexing for massive-scale multimodal single-cell sequencing.* Nat Methods, 2025. **22**(6): p. 1199-1212.
29. Kuijpers, L., et al., *Split Pool Ligation-based Single-cell Transcriptome sequencing (SPLiT-seq) data processing pipeline comparison.* BMC Genomics, 2024. **25**(1): p. 361.
30. Han, K.Y., et al., *SIDR: simultaneous isolation and parallel sequencing of genomic DNA and total RNA from single cells.* Genome Res, 2018. **28**(1): p. 75-87.
31. Chai, H., et al., *Tri-omic single-cell mapping of the 3D epigenome and transcriptome in whole mouse brains throughout the lifespan.* Nat Methods, 2025. **22**(5): p. 994-1007.
32. Lodato, M.A., et al., *Somatic mutation in single human neurons tracks developmental and transcriptional history.* Science, 2015. **350**(6256): p. 94-98.
33. Baldassari, S., et al., *Single-cell genotyping and transcriptomic profiling of mosaic focal cortical dysplasia.* Nat Neurosci, 2025. **28**(5): p. 964-972.
34. Wen, L. and F. Tang, *Recent advances in single-cell sequencing technologies.* Precis Clin Med, 2022. **5**(1): p. pbac002.
35. Chen, A., et al., *Spatiotemporal transcriptomic atlas of mouse organogenesis using DNA nanoball-patterned arrays.* Cell, 2022. **185**(10): p. 1777-1792 e21.
36. Zhao, Y., et al., *Stereo-seq V2: Spatial mapping of total RNA on FFPE sections with high resolution.* Cell, 2025.
37. Sun, E.D., et al., *Spatial transcriptomic clocks reveal cell proximity effects in brain ageing.* Nature, 2025. **638**(8049): p. 160-171.
38. Shi, H., et al., *Spatial atlas of the mouse central nervous system at molecular resolution.* Nature, 2023. **622**(7983): p. 552-561.

39. Maynard, K.R., et al., *Transcriptome-scale spatial gene expression in the human dorsolateral prefrontal cortex.* Nat Neurosci, 2021. **24**(3): p. 425-436.
40. Goltsev, Y., et al., *Deep Profiling of Mouse Splenic Architecture with CODEX Multiplexed Imaging.* Cell, 2018. **174**(4): p. 968-981 e15.
41. Black, S., et al., *CODEX multiplexed tissue imaging with DNA-conjugated antibodies.* Nat Protoc, 2021. **16**(8): p. 3802-3835.
42. Angelo, M., et al., *Multiplexed ion beam imaging of human breast tumors.* Nat Med, 2014. **20**(4): p. 436-42.
43. Yuan, Z., et al., *SEAM is a spatial single nuclear metabolomics method for dissecting tissue microenvironment.* Nat Methods, 2021. **18**(10): p. 1223-1232.
44. Zhang, D., et al., *Spatial epigenome-transcriptome co-profiling of mammalian tissues.* Nature, 2023. **616**(7955): p. 113-122.
45. Vickovic, S., et al., *SM-Omics is an automated platform for high-throughput spatial multi-omics.* Nat Commun, 2022. **13**(1): p. 795.
46. Liu, Y., et al., *High-Spatial-Resolution Multi-Omics Sequencing via Deterministic Barcoding in Tissue.* Cell, 2020. **183**(6): p. 1665-1681 e18.
47. Liu, Y., et al., *Spatial-CITE-seq: spatially resolved high-plex protein and whole transcriptome co-mapping.* Res Sq, 2022.
48. Deng, Y., et al., *Spatial profiling of chromatin accessibility in mouse and human tissues.* Nature, 2022. **609**(7926): p. 375-383.
49. Yang, A.C., et al., *A human brain vascular atlas reveals diverse mediators of Alzheimer's risk.* Nature, 2022. **603**(7903): p. 885-892.
50. Jorstad, N.L., et al., *Comparative transcriptomics reveals human-specific cortical features.* Science, 2023. **382**(6667): p. eade9516.
51. Braun, E., et al., *Comprehensive cell atlas of the first-trimester developing human brain.* Science, 2023. **382**(6667): p. eadf1226.
52. Kamath, T., et al., *Single-cell genomic profiling of human dopamine neurons identifies a population that selectively degenerates in Parkinson's disease.* Nat Neurosci, 2022. **25**(5): p. 588-595.
53. Agarwal, D., et al., *A single-cell atlas of the human substantia nigra reveals cell-specific pathways associated with neurological disorders.* Nat Commun, 2020. **11**(1): p. 4183.
54. Zhang, M., et al., *Molecularly defined and spatially resolved cell atlas of the whole mouse brain.* Nature, 2023. **624**(7991): p. 343-354.
55. Tian, W., et al., *Single-cell DNA methylation and 3D genome architecture in the human brain.* Science, 2023. **382**(6667): p. eadf5357.
56. Network, B.I.C.C., *A multimodal cell census and atlas of the mammalian primary motor cortex.* Nature, 2021. **598**(7879): p. 86-102.
57. Kozareva, V., et al., *A transcriptomic atlas of mouse cerebellar cortex comprehensively defines cell types.* Nature, 2021. **598**(7879): p. 214-219.
58. Yao, Z., et al., *A high-resolution transcriptomic and spatial atlas of cell types in the whole mouse brain.* Nature, 2023. **624**(7991): p. 317-332.
59. Walchli, T., et al., *Single-cell atlas of the human brain vasculature across development, adulthood and disease.* Nature, 2024. **632**(8025): p. 603-613.
60. Wang, P., et al., *Molecular pathways and diagnosis in spatially resolved Alzheimer's hippocampal atlas.* Neuron, 2025. **113**(13): p. 2123-2140 e9.

61. Murdock, M.H. and L.H. Tsai, *Insights into Alzheimer's disease from single-cell genomic approaches.* Nat Neurosci, 2023. **26**(2): p. 181-195.
62. von Maydell, D., et al., *ABCA7 variants impact phosphatidylcholine and mitochondria in neurons.* Nature, 2025.
63. Ardura-Fabregat, A., et al., *Response of spatially defined microglia states with distinct chromatin accessibility in a mouse model of Alzheimer's disease.* Nat Neurosci, 2025. **28**(8): p. 1688-1703.
64. Zeng, H., et al., *Integrative in situ mapping of single-cell transcriptional states and tissue histopathology in a mouse model of Alzheimer's disease.* Nat Neurosci, 2023. **26**(3): p. 430-446.
65. Gabitto, M.I., et al., *Integrated multimodal cell atlas of Alzheimer's disease.* Nat Neurosci, 2024. **27**(12): p. 2366-2383.
66. Gazestani, V., et al., *Early Alzheimer's disease pathology in human cortex involves transient cell states.* Cell, 2023. **186**(20): p. 4438-4453 e23.
67. Sun, N., et al., *Human microglial state dynamics in Alzheimer's disease progression.* Cell, 2023. **186**(20): p. 4386-4403 e29.
68. Hallacli, E., et al., *The Parkinson's disease protein alpha-synuclein is a modulator of processing bodies and mRNA stability.* Cell, 2022. **185**(12): p. 2035-2056 e33.
69. Garcia-Criado, F., et al., *Integrative Transcriptomic and Network-Based Analysis of Neuromuscular Diseases.* Int J Mol Sci, 2025. **26**(19).
70. Jin, Y., et al., *Whole-genome bisulfite sequencing of cell-free DNA unveils age-dependent and ALS-associated methylation alterations.* Cell Biosci, 2025. **15**(1): p. 26.
71. Haidet-Phillips, A.M., et al., *Astrocytes from familial and sporadic ALS patients are toxic to motor neurons.* Nat Biotechnol, 2011. **29**(9): p. 824-8.
72. Maniatis, S., et al., *Spatiotemporal dynamics of molecular pathology in amyotrophic lateral sclerosis.* Science, 2019. **364**(6435): p. 89-93.
73. Liu, Q., et al., *Single-cell, single-nucleus and xenium-based spatial transcriptomics analyses reveal inflammatory activation and altered cell interactions in the hippocampus in mice with temporal lobe epilepsy.* Biomark Res, 2024. **12**(1): p. 103.
74. Velmeshev, D., et al., *Single-cell genomics identifies cell type-specific molecular changes in autism.* Science, 2019. **364**(6441): p. 685-689.
75. Gui, J., et al., *Integrating Genetic and Single-Cell Genomic Data to Reveal Brain Cell-Specific Regulation of Attention-Deficit/Hyperactivity Disorder Risk in the Prefrontal Cortex.* Brain Behav, 2025. **15**(7): p. e70664.
76. Elkjaer, M.L., et al., *Single-Cell Multi-Omics Map of Cell Type-Specific Mechanistic Drivers of Multiple Sclerosis Lesions.* Neurol Neuroimmunol Neuroinflamm, 2024. **11**(3): p. e200213.
77. Han, B., et al., *Integrating spatial and single-cell transcriptomics to characterize the molecular and cellular architecture of the ischemic mouse brain.* Sci Transl Med, 2024. **16**(733): p. eadg1323.
78. Zhang, Y., et al., *Atlas of temporal molecular pathological alterations after traumatic brain injury based on RNA-Seq.* Exp Neurol, 2025. **390**: p. 115270.
79. Gao, J., et al., *Graph Neural Networks and Multimodal DTI Features for Schizophrenia Classification: Insights from Brain Network Analysis and Gene Expression.* Neurosci Bull, 2025. **41**(6): p. 933-950.
80. Wang, Z., et al., *Multiomics Analyses Reveal Microbiome-Gut-Brain Crosstalk Centered on Aberrant Gamma-Aminobutyric Acid and Tryptophan Metabolism in Drug-Naive Patients with First-Episode Schizophrenia.* Schizophr Bull, 2024. **50**(1): p. 187-198.

81. Mokhtari, A., et al., *Using multiomic integration to improve blood biomarkers of major depressive disorder: a case-control study.* EBioMedicine, 2025. **113**: p. 105569.
82. Li, Y., et al., *Multi-omics analysis of a drug-induced model of bipolar disorder in zebrafish.* iScience, 2023. **26**(5): p. 106744.
83. Bakken, T.E., et al., *Comparative cellular analysis of motor cortex in human, marmoset and mouse.* Nature, 2021. **598**(7879): p. 111-119.
84. Yao, Z., et al., *A transcriptomic and epigenomic cell atlas of the mouse primary motor cortex.* Nature, 2021. **598**(7879): p. 103-110.
85. Pollen, A.A., et al., *Molecular identity of human outer radial glia during cortical development.* Cell, 2015. **163**(1): p. 55-67.
86. Nowakowski, T.J., et al., *Spatiotemporal gene expression trajectories reveal developmental hierarchies of the human cortex.* Science, 2017. **358**(6368): p. 1318-1323.
87. Zhong, S., et al., *A single-cell RNA-seq survey of the developmental landscape of the human prefrontal cortex.* Nature, 2018. **555**(7697): p. 524-528.
88. Pollen, A.A., et al., *Establishing Cerebral Organoids as Models of Human-Specific Brain Evolution.* Cell, 2019. **176**(4): p. 743-756 e17.
89. Krienen, F.M., et al., *Innovations present in the primate interneuron repertoire.* Nature, 2020. **586**(7828): p. 262-269.
90. Hodge, R.D., et al., *Conserved cell types with divergent features in human versus mouse cortex.* Nature, 2019. **573**(7772): p. 61-68.
91. Jeong, H., et al., *Evolution of DNA methylation in the human brain.* Nat Commun, 2021. **12**(1): p. 2021.
92. Ziffra, R.S., et al., *Single-cell epigenomics reveals mechanisms of human cortical development.* Nature, 2021. **598**(7879): p. 205-213.
93. Zhou, Y., et al., *Human and mouse single-nucleus transcriptomics reveal TREM2-dependent and TREM2-independent cellular responses in Alzheimer's disease.* Nat Med, 2020. **26**(1): p. 131-142.
94. Mathys, H., et al., *Single-cell transcriptomic analysis of Alzheimer's disease.* Nature, 2019. **570**(7761): p. 332-337.
95. Fisher, E.M.C. and D.M. Bannerman, *Mouse models of neurodegeneration: Know your question, know your mouse.* Sci Transl Med, 2019. **11**(493).
96. Howe, J.R.t., et al., *The mouse as a model for neuropsychiatric drug development.* Curr Biol, 2018. **28**(17): p. R909-R914.
97. Pound, P. and M. Ritskes-Hoitinga, *Is it possible to overcome issues of external validity in preclinical animal research? Why most animal models are bound to fail.* J Transl Med, 2018. **16**(1): p. 304.
98. Roelfsema, P.R. and S. Treue, *Basic neuroscience research with nonhuman primates: a small but indispensable component of biomedical research.* Neuron, 2014. **82**(6): p. 1200-4.
99. Harding, J.D., *Nonhuman Primates and Translational Research: Progress, Opportunities, and Challenges.* ILAR J, 2017. **58**(2): p. 141-150.
100. Xu, T., et al., *Cross-species functional alignment reveals evolutionary hierarchy within the connectome.* Neuroimage, 2020. **223**: p. 117346.
101. Camp, J.G., et al., *Human cerebral organoids recapitulate gene expression programs of fetal neocortex development.* Proc Natl Acad Sci U S A, 2015. **112**(51): p. 15672-7.
102. Siletti, K., et al., *Transcriptomic diversity of cell types across the adult human brain.* Science, 2023. **382**(6667): p. eadd7046.

103. Maurano, M.T., et al., *Systematic localization of common disease-associated variation in regulatory DNA.* Science, 2012. **337**(6099): p. 1190-5.
104. Li, Q., et al., *Progress and opportunities of foundation models in bioinformatics.* Brief Bioinform, 2024. **25**(6).
105. Zhou, Z., et al. *DNABERT-2: Efficient Foundation Model and Benchmark For Multi-Species Genome.* 2023. arXiv:2306.15006 DOI: 10.48550/arXiv.2306.15006.
106. Nguyen, E., et al., *HyenaDNA: Long-Range Genomic Sequence Modeling at Single Nucleotide Resolution.* ArXiv, 2023.
107. Lin, A., et al., *Genos: a human-centric genomic foundation model.* Gigascience, 2025. **14**.
108. Brixi, G., et al., *Genome modelling and design across all domains of life with Evo 2.* Nature, 2026.
109. Avsec, Ž., et al., *Advancing regulatory variant effect prediction with AlphaGenome.* Nature, 2026. **649**(8099): p. 1206-1218.
110. Jumper, J., et al., *Highly accurate protein structure prediction with AlphaFold.* Nature, 2021. **596**(7873): p. 583-589.
111. Abramson, J., et al., *Accurate structure prediction of biomolecular interactions with AlphaFold 3.* Nature, 2024. **630**(8016): p. 493-500.
112. Frank, M., et al., *Leveraging a large language model to predict protein phase transition: A physical, multiscale, and interpretable approach.* Proc Natl Acad Sci U S A, 2024. **121**(33): p. e2320510121.
113. Cheng, J., et al., *Accurate proteome-wide missense variant effect prediction with AlphaMissense.* Science, 2023. **381**(6664): p. eadg7492.
114. Zeng, W.F., et al., *AlphaPeptDeep: a modular deep learning framework to predict peptide properties for proteomics.* Nat Commun, 2022. **13**(1): p. 7238.
115. Cui, H., et al., *scGPT: toward building a foundation model for single-cell multi-omics using generative AI.* Nat Methods, 2024. **21**(8): p. 1470-1480.
116. Theodoris, C.V., et al., *Transfer learning enables predictions in network biology.* Nature, 2023. **618**(7965): p. 616-624.
117. Hao, M., et al., *Large-scale foundation model on single-cell transcriptomics.* Nat Methods, 2024. **21**(8): p. 1481-1491.
118. Rosen, Y., et al., *Universal Cell Embeddings: A Foundation Model for Cell Biology.* 2024: p. 2023.11.28.568918.
119. Yang, X., et al., *GeneCompass: deciphering universal gene regulatory mechanisms with a knowledge-informed cross-species foundation model.* Cell Res, 2024. **34**(12): p. 830-845.
120. Zeng, Y., et al., *CellFM: a large-scale foundation model pre-trained on transcriptomics of 100 million human cells.* Nat Commun, 2025. **16**(1): p. 4679.
121. Ji, B., et al., *CAPTAIN: A multimodal foundation model pretrained on co-assayed single-cell RNA and protein.* 2025: p. 2025.07.07.663366.
122. Tejada-Lapuerta, A., et al., *Nicheformer: a foundation model for single-cell and spatial omics.* Nat Methods, 2025. **22**(12): p. 2525-2538.
123. Blampey, Q., et al., *Novae: a graph-based foundation model for spatial transcriptomics data.* Nat Methods, 2025. **22**(12): p. 2539-2550.
124. Chen, W., et al., *A visual-omics foundation model to bridge histopathology with spatial transcriptomics.* Nat Methods, 2025. **22**(7): p. 1568-1582.
125. Wang, C., et al., *scGPT-spatial: Continual Pretraining of Single-Cell Foundation Model for Spatial Transcriptomics.* 2025: p. 2025.02.05.636714.

126. Pang, J., et al., *OmniCell: Unified Foundation Modeling of Single-Cell and Spatial Transcriptomics for Cellular and Molecular Insights.* 2025: p. 2025.12.29.696804.

127. Singhal, K., et al. *Towards Expert-Level Medical Question Answering with Large Language Models*. 2023. arXiv:2305.09617 DOI: 10.48550/arXiv.2305.09617.

128. Yang, X., et al., *GatorTron: A Large Language Model for Clinical Natural Language Processing.* 2022: p. 2022.02.27.22271257.

129. Peng, C., et al., *A study of generative large language model for medical research and healthcare.* NPJ Digit Med, 2023. **6**(1): p. 210.

130. Chen, Z., et al. *MEDITRON-70B: Scaling Medical Pretraining for Large Language Models*. 2023. arXiv:2311.16079 DOI: 10.48550/arXiv.2311.16079.

131. Nguyen, E., et al., *Sequence modeling and design from molecular to genome scale with Evo.* Science, 2024. **386**(6723): p. eado9336.

132. Santuz, H., et al., *Small Oligomers of Aβ42 Protein in the Bulk Solution with AlphaFold2.* ACS Chem Neurosci, 2022. **13**(6): p. 711-713.

133. Lin, Z., et al., *Evolutionary-scale prediction of atomic-level protein structure with a language model.* Science, 2023. **379**(6637): p. 1123-1130.

134. Gessulat, S., et al., *Prosit: proteome-wide prediction of peptide tandem mass spectra by deep learning.* Nat Methods, 2019. **16**(6): p. 509-518.

135. Bouwmeester, R., et al., *DeepLC can predict retention times for peptides that carry as-yet unseen modifications.* Nat Methods, 2021. **18**(11): p. 1363-1369.

136. Yan, C., et al., *Leveraging generative AI to prioritize drug repurposing candidates for Alzheimer's disease with real-world clinical validation.* NPJ Digit Med, 2024. **7**(1): p. 46.

137. Xiong, L.L., et al., *Single-cell RNA sequencing reveals B cell-related molecular biomarkers for Alzheimer's disease.* Exp Mol Med, 2021. **53**(12): p. 1888-1901.

138. Xu, H. and J. Jia, *Single-Cell RNA Sequencing of Peripheral Blood Reveals Immune Cell Signatures in Alzheimer's Disease.* Front Immunol, 2021. **12**: p. 645666.

139. Patel, A.P., et al., *Single-cell RNA-seq highlights intratumoral heterogeneity in primary glioblastoma.* Science, 2014. **344**(6190): p. 1396-401.

140. Couturier, C.P., et al., *Single-cell RNA-seq reveals that glioblastoma recapitulates a normal neurodevelopmental hierarchy.* Nat Commun, 2020. **11**(1): p. 3406.

141. Paananen, J. and V. Fortino, *An omics perspective on drug target discovery platforms.* Brief Bioinform, 2020. **21**(6): p. 1937-1953.

142. Yi, G., et al., *Integration of multi-omics transcriptome-wide analysis for the identification of novel therapeutic drug targets in diabetic retinopathy.* J Transl Med, 2024. **22**(1): p. 1146.

143. Badia, I.M.P., et al., *Gene regulatory network inference in the era of single-cell multi-omics.* Nat Rev Genet, 2023. **24**(11): p. 739-754.

144. Jain, S., et al., *Single-cell RNA sequencing and spatial transcriptomics reveal cancer-associated fibroblasts in glioblastoma with protumoral effects.* J Clin Invest, 2023. **133**(5).

145. Le, J., et al., *Single-cell multi-omics in cancer immunotherapy: from tumor heterogeneity to personalized precision treatment.* Mol Cancer, 2025. **24**(1): p. 221.

146. Ahmed, Z., *Practicing precision medicine with intelligently integrative clinical and multi-omics data analysis.* Hum Genomics, 2020. **14**(1): p. 35.

147. Perdyan, A., et al., *Integration of single-cell RNA sequencing and spatial transcriptomics to reveal the glioblastoma heterogeneity.* F1000Res, 2022. **11**: p. 1180.

148. Haghverdi, L., et al., *Batch effects in single-cell RNA-sequencing data are corrected by matching mutual nearest neighbors.* Nat Biotechnol, 2018. **36**(5): p. 421-427.

149. Tran, H.T.N., et al., *A benchmark of batch-effect correction methods for single-cell RNA sequencing data.* Genome Biol, 2020. **21**(1): p. 12.

150. Hrovatin, K., et al., *Integrating single-cell RNA-seq datasets with substantial batch effects.* bioRxiv, 2024.

151. Regev, A., et al., *The Human Cell Atlas.* Elife, 2017. **6**.

152. Park, S.H., J. Choi, and J.S. Byeon, *Key Principles of Clinical Validation, Device Approval, and Insurance Coverage Decisions of Artificial Intelligence.* Korean J Radiol, 2021. **22**(3): p. 442-453.

153. Li, M.M., K. Huang, and M. Zitnik, *Graph representation learning in biomedicine and healthcare.* Nat Biomed Eng, 2022. **6**(12): p. 1353-1369.

154. Tang, X., et al., *The single-cell sequencing: new developments and medical applications.* Cell Biosci, 2019. **9**: p. 53.

155. Landry, A.P., et al., *Establishing the utility of multi-platform liquid biopsy by integrating the CSF methylome and proteome in CNS tumours.* J Neurooncol, 2024. **169**(2): p. 233-239.

156. Di Sario, G., et al., *Enhancing clinical potential of liquid biopsy through a multi-omic approach: A systematic review.* Front Genet, 2023. **14**: p. 1152470.

157. Hawrylycz, M., et al., *A guide to the BRAIN Initiative Cell Census Network data ecosystem.* PLoS Biol, 2023. **21**(6): p. e3002133.

158. Amunts, K., et al., *The Human Brain Project: Creating a European Research Infrastructure to Decode the Human Brain.* Neuron, 2016. **92**(3): p. 574-581.

159. Poo, M.M., et al., *China Brain Project: Basic Neuroscience, Brain Diseases, and Brain-Inspired Computing.* Neuron, 2016. **92**(3): p. 591-596.

160. Okano, H., A. Miyawaki, and K. Kasai, *Brain/MINDS: brain-mapping project in Japan.* Philos Trans R Soc Lond B Biol Sci, 2015. **370**(1668).
