# Supplementary material for: From the brain cell atlas to precision neurology: a review of the application of AI-driven multi-omics in brain science
Source: Gigascience. 2026 Jun 23;15:giag075. doi: 10.1093/gigascience/giag075 (PMC13354932; doi:10.1093/gigascience/giag075)
Supplement: giag075_Authors_Response_To_Reviewer_Comments_Original_Submission [file giag075_authors_response_to_reviewer_comments_original_submission.pdf]

Author's Response To Reviewer Comments

Dear Dr. Ma and esteemed reviewers,
Thank you for the opportunity to revise our manuscript entitled "From the Brain Cell Atlas to Precision Neurology: A review of the application of AI-driven multi-omics in brain science" (Manuscript ID: GIGA-D-26-00090). We sincerely appreciate the time and effort that you and the reviewers have dedicated to evaluating our work. We are especially grateful for the constructive and insightful comments, which have helped improve the clarity, scientific rigor, and overall quality of the study.
In the revised manuscript, we have addressed all reviewer comments point by point and have made substantial revisions throughout the manuscript. In particular, we have added comprehensive reference tables summarizing existing healthy, disease, and cross-species brain atlases, alongside benchmarking insights for current AI models. Furthermore, we have introduced dedicated sections to critically discuss the inherent trade-offs of spatial modalities, the boundaries between cell types and cell states, the computational uncertainties of mapping diseased cells to healthy references, and the emerging paradigm of neurological digital twins.
For ease of reference, the editor's and reviewers' comments are shown in bold, our responses are provided in italics, and the corresponding revisions in the manuscript are highlighted in blue.
We hope that the revised manuscript is now suitable for consideration for publication in GigaScience, and we thank you again for your time and attention.
Yours sincerely,
Lifang Wang on behalf of all co-authors
Key Laboratory of Brain Cell Mapping of Zhejiang Province, BGI Research, Hangzhou, Zhejiang 310030, China
E-mail: wanglifang@genomics.cn

Reviewer reports:

Reviewer #1: Advances in brain cell atlasing is an interesting and timely perspective to be revised as it serves as the essential data foundation for AI-driven precision neurology. However, to highlight the beneficial role of the multi-omics construction of next generation brain atlases in neurology, the authors should expand the revision of those healthy, disease, single and multi-species brain atlases already existing. I have some major and minor concerns about the manuscript, as follows:

Major comments:
1.The authors should add tables summarizing the healthy, disease, single and multi-species brain atlases revised, expanding them in number and features. It could be useful, for example, to know if their data are publicly available or not, what are their pro and cons in the perspective of obtaining a "multi-omics construction of next generation of brain atlases in neurology" from single cell and spatial studies/projects.

Response：We thank the reviewer for this helpful suggestion. In the revised manuscript, we added a new table summarizing representative healthy/reference brain atlases, disease-focused brain atlases, and single-species versus multi-species brain atlas resources (Table S1). These tables now include species, brain region, disease context, omics modality, spatial information, data availability, major findings, strengths, limitations, and relevance to next-generation multi-omics atlas construction in neurology. We also added a synthesis paragraph comparing the pros and cons of single-cell, spatial, multi-omics, single-species, and multi-species atlas strategies. These revisions make the manuscript more useful as a structured resource for readers and clarify how different atlas types contribute to precision neurology (see lines 250-263，369-374，456-460).

2.Data standardization and ethical concerns are themes only superficially reported.

Response：We thank the reviewer for this important observation. To move beyond a superficial reporting of these themes, we have substantially expanded the section “From laboratory to clinical: challenges and perspectives” by introducing two dedicated subsections: “Data Standardization and Harmonization” and “Ethical Considerations in Large-Scale Brain Multi-Omics Research”
In the “Data Standardization and Harmonization” subsection, we critically dissect the technical bottlenecks of multi-omics integration, including batch effects, cross-platform technical variability, and discrepancies in sample processing/computational pipelines. We highlight how major international initiatives (such as the Human Cell Atlas and the BRAIN Initiative) are actively driving unified quality-control metrics, standardized metadata schemas. Meanwhile, we enphasize the imperative of adhering to the FAIR data principles to enable global collaborative workflows. In the “Ethical Considerations in Large-Scale Brain Multi-Omics Research” subsection, we have expanded the discussion to address informed consent, privacy protection, controlled-access data sharing, equity and representation in global atlas construction, and the ethical implications of AI-driven diagnostic tools in neurological settings, including algorithmic bias, external validation, clinician oversight, and regulatory monitoring (see lines 912-956).

Minor:
1.Lines 37-38 at page five refer to the brain atlas without any reference. It is not clear if it is cited as a resource or as a research goal. Please, clarify this point.

Response：Thank you for this helpful comment. We agree that the phrase “the brain atlas” was not sufficiently clear and might imply a specific atlas resource. Our intention was to refer to brain atlases as a general class of reference resources generated through brain mapping studies, rather than to cite one particular atlas. We have therefore revised the sentence to avoid ambiguity and added relevant references to representative brain atlas studies (see lines 39-45).

2.Please, check the text for missing punctuation.

Response：We thank the reviewer for this careful reading. We have conducted a thorough proofreading of the entire manuscript and corrected all identified instances of missing punctuation, including missing periods at sentence ends, missing commas in enumerations, and inconsistent punctuation following figure legends and section headings.

3.Digital twins use is emerging as a resource and could be also discussed.

Response：We thank the reviewer for this helpful suggestion. We agree that digital twins should not be presented only as a speculative future concept, because disease-specific applications have already begun to emerge in neurological research. We have therefore revised the “Looking ahead: The next decade of brain disease research” section to include a more literature-grounded discussion of neurological digital twins, with representative examples from epilepsy and Alzheimer’s disease (see lines 889-901).

Reviewer #2: In the manuscript titled "From the Brain Cell Atlas to Precision Neurology," He et al. cover a timely area and pull together a broad literature on brain atlases, multi-omics, AI models, and disease. The breadth is useful, but the paper is structured more as a survey of methods and future directions than a critical review of what these approaches have established in brain biology and disease.

Major comments:
1. The introduction and early technology sections need a stronger conceptual grounding. The paper opens by outlining the shift from descriptive atlas building to mechanism and precision neurology, but then becomes largely a tour of platforms, from scRNA-seq to Stereo-seq and MERFISH. What does each modality uniquely contribute in brain tissue, what information is lost at each step, and what kinds of biological claims can each modality support?

Response：We thank the reviewer for this substantive and helpful critique. To adress this, we have throughly revised both the Introduction and the early “Multi-omics mapping technology platform” section to provide a stronger conceptual framework.
In the revised Introduction, we now frame the review around a paradigm shift from descriptive atlas

construction to mechanism-driven, AI-enabled precision neurology. Specifically, we clarify that modern brain atlases are not merely anatomical or molecular maps, but reference systems for linking cellular identity, spatial organization, cross-species conservation, disease-associated perturbations and clinical stratification. We also reorganized the opening logic around two complementary forces: first, emerging single-cell, spatial multi-omics and AI technologies that enable mechanistic interpretation of brain heterogeneity; and second, the evolution of atlas-based research goals from describing “what the brain looks like” toward explaining “how it works” and “how it goes awry” in disease (see lines46-78).

In the revised technology section, we further address the reviewer’s specific concern by discussing each major modality in terms of three questions: what biological information it uniquely provides in brain tissue, what information is lost or compromised, and what types of biological claims it can support. For example, we now clarify that scRNA-seq and snRNA-seq support high-throughput cell-type and cell-state discovery but lose native spatial context; spatial transcriptomic methods preserve anatomical organization but trade off sensitivity, throughput or gene coverage depending on the platform; and joint multi-omics approaches improve mechanistic inference but remain constrained by lower depth, higher technical complexity and limited scalability. These conceptual distinctions are also summarized in the revised Table 1 (see lines101-170)

.

2. Since the review is framed around the brain cell atlas, it should directly address instability of cell type definitions across studies, region-specific versus whole brain taxonomies, cell type versus cell state, and uncertainty when mapping diseased cells onto healthy references. These issues are especially important given the paper's movement from healthy atlas examples such as cortex, hippocampus, and fetal brain to later claims about disease mechanisms and precision neurology.

Response：We are deeply grateful to the reviewer for pointing out this critical conceptual gap. To adress this comprehensively, we have introduced a brand-new, dedicated subsection titled"II. Conceptual and computational caveats in brain cell atlas interpretation" postioned precisely between the healthy reference and disease sections.

This subsection systematically unpackes the four core challenges raised by the reviewer: 1) instability of cell type definitions: we discuss how technical and algorithmic variations (e.g., dissociation protocols, sequencing depth, clustering resolutions) lead to discordanct taxonomies. We emphasize the cell labels must be treated as hierarchical, context-dependent annotations rather than absolute biological entities, and advocate for validation via multi-modal evidence. 2) region-specific versus whole-brain taxonomies: We contrast the macro-scale harmonized frameworks of whole-brain atlases with the high-precision localized specialization captured by region-specific studies. We explicitly discuss why findings from specific regions (e.g., cortex, hippocampus, cerebellum, or fetal brain) cannot be generalized to the entire organ or all neurological disorders without accounting for spatial and developmental contexts. 3) cell type versus cell state: we clearly delineate the conceptual boundaries between stable, lineage-determined cell types and dynamic, transient, or reversible cell states induced by microenvironmental perturbations, noting that over-interpreting states as new types can obfuscate primary disease drivers. 4) uncertainty in disease-to-healthy mapping: we critically analyze the computational risks of "out-of-distribution" label transfer, where altered or unique pathological cells are artificially forced into healthy categories. We frame reference mapping as a hypothesis-generating strategy rather than definitive proof, and outline necessary safeguards, including label confidence scores, out-of-distribution detection, and mandatory disease-specific or histopathological validation. (see lines 264--309).

3. The disease and cross-species sections should describe replication across studies, shared features across disorders, and robustness of cross-species alignment for finer cell types. It would be helpful to highlight a few biological themes, such as selective cellular vulnerability, glial activation, or developmental dysregulation, and describing what is known about feature conservation between human and model species.

Response：We thank the reviewer for this helpful comment. We have revised both the disease and cross-species sections to provide a more integrative discussion. In the disease section, we added a new synthesis paragraph summarizing replicated and shared cellular programs across disorders, including selective neuronal vulnerability, glial activation and state transitions, and developmental or maturation-related dysregulation. We also clarified that replication is generally stronger at the level of broad cell classes and pathways than at the level of individual marker genes or highly resolved disease-associated cell states (see

lines 375-419).
In the cross-species section, we added a new discussion on the robustness and limitations of cross-species cell-type alignment. We now distinguish relatively robust alignment of major cell classes from less stable alignment of fine neuronal subtypes, transient developmental populations, and disease-associated glial states. We further discuss which molecular features are more conserved across species, such as canonical cell identity markers and core pathways, and which are more species-specific, such as enhancer usage, chromatin accessibility, cell-type proportions, developmental timing, and disease-induced cell states. We also added a summary Table2 to highlight these themes and their translational implications (see lines 461-489).

4. The AI section lists an inventory of cell foundation and spatial models without providing the reader guidance in selecting a model for different applications. For example, some models have been trained primarily using bulk or non-brain tissue genomics data. Table 2 should include more information about benchmarking on brain datasets and major limitations.

Response：We appreciate this targeted critique of Table 3. We have substantially revised the table to include two additional columns: "Brain Dataset Benchmarking" (specifying which brain-relevant datasets each model has been evaluated on, if any) and "Key Limitations for Brain Applications" (summarizing major known shortcomings). We also added narrative text within the AI section explicitly guiding readers on model selection criteria for different brain research applications: for example, recommending scGPT or CellFM for cell type annotation tasks with large training set coverage, Geneformer for in silico perturbation modeling where experimental data are unavailable, and spatial foundation models such as OmniCell for tasks requiring preservation of tissue architecture. We also note that several models (e.g., scFoundation, UCE) were primarily trained on non-brain or bulk tissue data, and their performance on brain-specific rare cell populations should be independently validated before deployment (see lines519-528).

5. The review often moves too quickly from atlas associations or model capability to disease stratification, biomarker discovery, and therapeutic targets. Statements about "unprecedented" analytical power, near-term clinical deployment, and implementation within five to ten years should cite supporting evidence and potential roadblocks. There should be more specific discussion of how ICPBM relates to other large, collaborative efforts to study brain function and disease.

Response：We thank the reviewer for this important point regarding the gap between analytical capability and clinical readiness. We have revised relevant sections to (1) temper unqualified claims of "unprecedented" power with specific citations and caveats about current limitations; (2) add explicit discussion of the roadblocks to five-to-ten year clinical implementation, including data governance, prospective validation requirements, reimbursement frameworks, and the need for regulatory-grade evidence; and (3) expand the ICPBM discussion to situate it explicitly in relation to BICCN, the Human Brain Project, China Brain Project, and Brain/MINDS, clarifying how ICPBM's focus on primate multi-omics and 25-year timeline complements rather than duplicates these existing efforts (see lines 663-671, 711-718, 845-849, 871-877, 957-985).

To help contextualize the literature background and align with recent developments in the field, the following articles published in GigaScience may be of interest in relation to your review:
(1) Fabian Hausmann, Lucas Caldi Gomes, Sonja Hänzelmann, Robin Khatri, Sergio Oller, Marie Gebelin, Mojan Parvaz, Laura Tzeplaeff, Laura Pasetto, Qihui Zhou, Pavol Zelina, Dieter Edbauer, R Jeroen Pasterkamp, Hubert Rehrauer, Ralph Schlapbach, Christine Carapito, Valentina Bonetto, Stefan Bonn, Paul Lingor, A dataset profiling the multiomic landscape of the prefrontal cortex in amyotrophic lateral sclerosis, GigaScience, Volume 13, 2024, giae100, https://doi.org/10.1093/gigascience/giae100
(2) Chao Zhang, Lin Liu, Ying Zhang, Mei Li, Shuangsang Fang, Qiang Kang, Ao Chen, Xun Xu, Yong Zhang, Yuxiang Li, spatiAlign: an unsupervised contrastive learning model for data integration of spatially resolved transcriptomics, GigaScience, Volume 13, 2024, giae042, https://doi.org/10.1093/gigascience/giae042
(3) Xiuyun Liu, Fangfang Li, Marek Czosnyka, Zofia Czosnyka, Huijie Yu, Xiaoguang Tong, Yan Xing, Hongliang Li, Ke Pu, Keke Feng, Kuo Zhang, Meijun Pang, Dong Ming, Multi-omics and high-spatial-resolution omics: deciphering complexity in neurological disorders, GigaScience, Volume 14, 2025, giaf137,

https://doi.org/10.1093/gigascience/giaf137

Response : We thank the reviewers for recommending these highly relevant publications from GigaScience. We have incorporated all three references into the revised manuscript at appropriate locations [28,136,250].

Editorial Manager®

Cookie Preference Center

We use cookies which are necessary to make our site work. We may also use additional cookies to analyze, improve and personalize our content and your digital experience. For more information, see our Cookie Policy.

You may choose not to allow some types of cookies. However, blocking some types may impact your experience of our site and the services we are able to offer. See the different category headings below to find out more or change your settings.

Allow all

Manage Consent Preferences

Strictly Necessary Cookies

Always active

These cookies are necessary for the website to function and cannot be switched off in our systems. They are usually only set in response to actions made by you which amount to a request for services, such as setting your privacy preferences, logging in or filling in forms. You can set your browser to block or alert you about these cookies, but some parts of the site will not then work.

Functional Cookies

These cookies enable the website to provide enhanced functionality and personalisation. They may be set by us or by third party providers whose services we

have added to our pages. If you do not allow these cookies then some or all of these services may not function properly.

Performance Cookies

These cookies allow us to count visits and traffic sources so we can measure and improve the performance of our site. They help us to know which pages are the most and least popular and see how visitors move around the site.

Targeting Cookies

These cookies may be set through our site by our advertising partners. They may be used by those companies to build a profile of your interests and show you relevant adverts on other sites. If you do not allow these cookies, you will experience less targeted advertising.

Cookie List

Clear

Apply Cancel

Consent Leg.Interest

Confirm my choices

Power
