# Supplementary material for: From the brain cell atlas to precision neurology: a review of the application of AI-driven multi-omics in brain science
Source: Gigascience. 2026 Jun 23;15:giag075. doi: 10.1093/gigascience/giag075 (PMC13354932; doi:10.1093/gigascience/giag075)
Supplement: giag075_Reviewer_1_Report_Original_Submission — Reviewer 1 -- 4/13/2026 [file giag075_reviewer_1_report_original_submission.pdf]

Reviewer Report

Title: From the Brain Cell Atlas to Precision Neurology: A review of the application of AI-driven multi-omics in brain science

Version: Original Submission **Date:** 4/13/2026

Reviewer name: Reviewer 1

Reviewer Comments to Author:

Advances in brain cell atlasing is an interesting and timely perspective to be revised as it serves as the essential data foundation for AI-driven precision neurology. However, to highlight the beneficial role of the multi-omics construction of next generation brain atlases in neurology, the authors should expand the revision of those healthy, disease, single and multi-species brain atlases already existing. I have some major and minor concerns about the manuscript, as follows:Major:- The authors should add tables summarizing the healthy, disease, single and multi-species brain atlases revised, expanding them in number and features. It could be useful, for example, to know if their data are publicly available or not, what are their pro and cons in the perspective of obtaining a "multi-omics construction of next generation of brain atlases in neurology" from single cell and spatial studies/projects.- Data standardization and ethical concerns are themes only superficially reported.Minor:- Lines 37-38 at page five refer to the brain atlas without any reference. It is not clear if it is cited as a resource or as a research goal. Please, clarify this point.- Please, check the text for missing punctuation.- Digital twins use is emerging as a resource and could be also discussed.

Level of Interest

Please indicate how interesting you found the manuscript: Choose an item.

Quality of Written English

Please indicate the quality of language in the manuscript: Choose an item.

Declaration of Competing Interests

Please complete a declaration of competing interests, considering the following questions:

- Have you in the past five years received reimbursements, fees, funding, or salary from an organisation that may in any way gain or lose financially from the publication of this manuscript, either now or in the future?
- Do you hold any stocks or shares in an organisation that may in any way gain or lose financially from the publication of this manuscript, either now or in the future?
- Do you hold or are you currently applying for any patents relating to the content of the manuscript?

- Have you received reimbursements, fees, funding, or salary from an organization that holds or has applied for patents relating to the content of the manuscript?
- Do you have any other financial competing interests?
- Do you have any non-financial competing interests in relation to this paper?

If you can answer no to all of the above, write 'I declare that I have no competing interests' below. If your reply is yes to any, please give details below.

I declare that I have no competing interests' below

I agree to the open peer review policy of the journal. I understand that my name will be included on my report to the authors and, if the manuscript is accepted for publication, my named report including any attachments I upload will be posted on the website along with the authors' responses. I agree for my report to be made available under an Open Access Creative Commons CC-BY license (http://creativecommons.org/licenses/by/4.0/). I understand that any comments which I do not wish to be included in my named report can be included as confidential comments to the editors, which will not be published.

I agree to the open peer review policy of the journal

To further support our reviewers, we have joined with Publons, where you can gain additional credit to further highlight your hard work (see: https://publons.com/journal/530/gigascience). On publication of this paper, your review will be automatically added to Publons, you can then choose whether or not to claim your Publons credit. I understand this statement.

Yes Choose an item.
