# Supplementary material for: From the brain cell atlas to precision neurology: a review of the application of AI-driven multi-omics in brain science
Source: Gigascience. 2026 Jun 23;15:giag075. doi: 10.1093/gigascience/giag075 (PMC13354932; doi:10.1093/gigascience/giag075)
Supplement: giag075_Reviewer_2_Report_Original_Submission — Reviewer 2 -- 4/20/2026 [file giag075_reviewer_2_report_original_submission.pdf]

Reviewer Report

Title: From the Brain Cell Atlas to Precision Neurology: A review of the application of AI-driven multi-omics in brain science

Version: Original Submission **Date:** 4/20/2026

Reviewer name: Reviewer 2

Reviewer Comments to Author:

In the manuscript titled "From the Brain Cell Atlas to Precision Neurology," He et al. cover a timely area and pull together a broad literature on brain atlases, multi-omics, AI models, and disease. The breadth is useful, but the paper is structured more as a survey of methods and future directions than a critical review of what these approaches have established in brain biology and disease.Major comments:1. The introduction and early technology sections need a stronger conceptual grounding. The paper opens by outlining the shift from descriptive atlas building to mechanism and precision neurology, but then becomes largely a tour of platforms, from scRNA-seq to Stereo-seq and MERFISH. What does each modality uniquely contribute in brain tissue, what information is lost at each step, and what kinds of biological claims can each modality support?2. Since the review is framed around the brain cell atlas, it should directly address instability of cell type definitions across studies, region-specific versus whole brain taxonomies, cell type versus cell state, and uncertainty when mapping diseased cells onto healthy references. These issues are especially important given the paper's movement from healthy atlas examples such as cortex, hippocampus, and fetal brain to later claims about disease mechanisms and precision neurology.3. The disease and cross-species sections should describe replication across studies, shared features across disorders, and robustness of cross-species alignment for finer cell types. It would be helpful to highlight a few biological themes, such as selective cellular vulnerability, glial activation, or developmental dysregulation, and describing what is known about feature conservation between human and model species.4. The AI section lists an inventory of cell foundation and spatial models without providing the reader guidance in selecting a model for different applications. For example, some models have been trained primarily using bulk or non-brain tissue genomics data. Table 2 should include more information about benchmarking on brain datasets and major limitations.5. The review often moves too quickly from atlas associations or model capability to disease stratification, biomarker discovery, and therapeutic targets. Statements about "unprecedented" analytical power, near-term clinical deployment, and implementation within five to ten years should cite supporting evidence and potential roadblocks. There should be more specific discussion of how ICPBM relates to other large, collaborative efforts to study brain function and disease.

Level of Interest

Please indicate how interesting you found the manuscript: Choose an item.

Quality of Written English

Please indicate the quality of language in the manuscript: Choose an item.

Declaration of Competing Interests

Please complete a declaration of competing interests, considering the following questions:

- Have you in the past five years received reimbursements, fees, funding, or salary from an organisation that may in any way gain or lose financially from the publication of this manuscript, either now or in the future?
- Do you hold any stocks or shares in an organisation that may in any way gain or lose financially from the publication of this manuscript, either now or in the future?
- Do you hold or are you currently applying for any patents relating to the content of the manuscript?
- Have you received reimbursements, fees, funding, or salary from an organization that holds or has applied for patents relating to the content of the manuscript?
- Do you have any other financial competing interests?
- Do you have any non-financial competing interests in relation to this paper?

If you can answer no to all of the above, write 'I declare that I have no competing interests' below. If your reply is yes to any, please give details below.

I declare that I have no competing interests.

I agree to the open peer review policy of the journal. I understand that my name will be included on my report to the authors and, if the manuscript is accepted for publication, my named report including any attachments I upload will be posted on the website along with the authors' responses. I agree for my report to be made available under an Open Access Creative Commons CC-BY license (http://creativecommons.org/licenses/by/4.0/). I understand that any comments which I do not wish to be included in my named report can be included as confidential comments to the editors, which will not be published.

I agree to the open peer review policy of the journal

To further support our reviewers, we have joined with Publons, where you can gain additional credit to further highlight your hard work (see: https://publons.com/journal/530/gigascience). On publication of this paper, your review will be automatically added to Publons, you can then choose whether or not to claim your Publons credit. I understand this statement.

Yes Choose an item.
